# Regulation of carotenogenesis in the red yeast *Xanthophyllomyces dendrorhous*: the role of the transcriptional co-repressor complex Cyc8–Tup1 involved in catabolic repression

**DOI:** 10.1186/s12934-016-0597-1

**Published:** 2016-11-14

**Authors:** Pamela Córdova, Jennifer Alcaíno, Natalia Bravo, Salvador Barahona, Dionisia Sepúlveda, María Fernández-Lobato, Marcelo Baeza, Víctor Cifuentes

**Affiliations:** 1 Departamento de Ciencias Ecológicas, Facultad de Ciencias, Universidad de Chile, Las Palmeras 3425, Casilla 653, Ñuñoa, Santiago, Chile; 2 Centro de Biología Molecular Severo Ochoa, Departamento de Biología Molecular (UAM-CSIC), Universidad Autónoma Madrid, Campus de Cantoblanco, calle Nicolás Cabrera No 1, Cantoblanco, 28049 Madrid, Spain

**Keywords:** Catabolic repression, Cyc8–Tup1 co-repressor complex, Carotenogenesis, Transcriptional regulation, *Xanthophyllomyces dendrorhous*

## Abstract

**Background:**

The yeast *Xanthophyllomyces dendrorhous* produces carotenoids of commercial interest, including astaxanthin and β-carotene. Although carotenogenesis in this yeast and the expression profiles of the genes controlling this pathway are known, the mechanisms regulating this process remain poorly understood. Several studies have demonstrated that glucose represses carotenogenesis in *X. dendrorhous*, suggesting that this pathway could be regulated by catabolic repression. Catabolic repression is a highly conserved regulatory mechanism in eukaryotes and has been widely studied in *Saccharomyces cerevisiae*. Glucose-dependent repression is mainly observed at the transcriptional level and depends on the DNA-binding regulator Mig1, which recruits the co-repressor complex Cyc8–Tup1, which then represses the expression of target genes. In this work, we studied the regulation of carotenogenesis by catabolic repression in *X. dendrorhous*, focusing on the role of the co-repressor complex Cyc8–Tup1.

**Results:**

The *X. dendrorhous CYC8* and *TUP1* genes were identified, and their functions were demonstrated by heterologous complementation in *S. cerevisiae.* In addition, *cyc8* ^−^ and *tup1* ^−^ mutant strains of *X. dendrorhous* were obtained, and both mutations were shown to prevent the glucose-dependent repression of carotenogenesis in *X. dendrorhous*, increasing the carotenoid production in both mutant strains. Furthermore, the effects of glucose on the transcript levels of genes involved in carotenogenesis differed between the mutant strains and wild-type *X. dendrorhous*, particularly for genes involved in the synthesis of carotenoid precursors, such as *HMGR*, *idi* and *FPS*. Additionally, transcriptomic analyses showed that *cyc8* ^−^ and *tup1* ^−^ mutations affected the expression of over 250 genes in *X. dendrorhous.*

**Conclusions:**

The *CYC8* and *TUP1* genes are functional in *X. dendrorhous*, and their gene products are involved in catabolic repression and carotenogenesis regulation. This study presents the first report involving the participation of Cyc8 and Tup1 in carotenogenesis regulation in yeast.

**Electronic supplementary material:**

The online version of this article (doi:10.1186/s12934-016-0597-1) contains supplementary material, which is available to authorized users.

## Background

For decades, the carotenogenic yeast *Xanthophyllomyces dendrorhous* has been widely studied due to its natural ability to synthesize astaxanthin, a carotenoid of commercial interest with uses in the aquaculture, food, pharmaceutical and cosmetics industries [1, 2]. Over time, various *X. dendrorhous* isolates have been obtained from cold regions around the world. This yeast was originally isolated from tree exudates in mountainous regions of Japan and Alaska [3]; more recently, isolates have been obtained from the Argentinian Patagonia [4], the southern region of Chile [5] and the sub-Antarctic region [6].

**Fig. 1:**
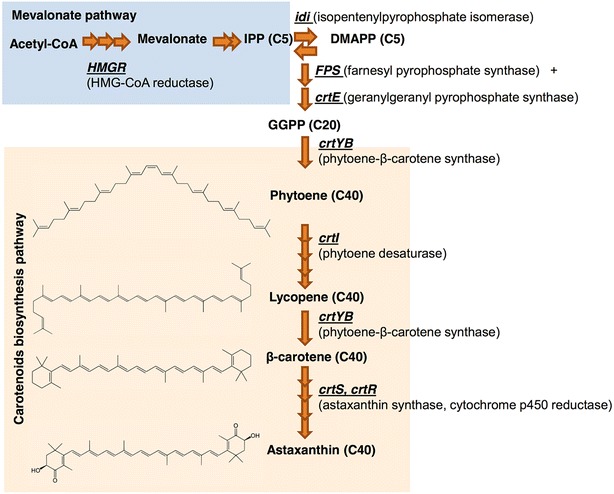
Overview of *X. dendrorhous* carotenogenesis. The genes (*underlined*) and enzymes (in *parentheses*) involved in each step are shown. Steps exclusive to the “mevalonate pathway” and “carotenoid biosynthesis” are enclosed in* blue* and* orange squares*, respectively. Structures of carotenoids are shown at the* left*. *IPP* isopentenyl pyrophosphate, *DMAPP* dimethylallyl pyrophosphate, *GGPP* geranylgeranyl pyrophosphate. *Cn* the number (n) of carbon atoms in each molecule

Many studies have attempted to improve astaxanthin production in *X. dendrorhous* (reviewed in [2]), contributing to our current knowledge of the genetic control of carotenogenesis in this yeast (Fig. 1). As in other non-photosynthetic eukaryotes, carotenoid synthesis in *X. dendrorhous* derives from the mevalonate pathway [2, 7, 8], which produces isopentenyl pyrophosphate (IPP). IPP is isomerized to dimethylallyl pyrophosphate (DMAPP) by the enzyme IPP isomerase, encoded by the *idi* gene [9]. Then, a molecule of DMAPP is sequentially condensed with three molecules of IPP to generate geranylgeranyl pyrophosphate (GGPP); these steps involve the prenyl transferase enzymes farnesyl pyrophosphate synthase and geranylgeranyl pyrophosphate synthase, encoded by the *FPS* and *crtE* genes, respectively [10]. Phytoene is the first carotenoid synthesized in the pathway, produced from GGPP by the bifunctional enzyme phytoene-β-carotene synthase (PBS, encoded by the *crtYB* gene) [11]. Then, phytoene is transformed to lycopene by phytoene desaturase (encoded by the *crtI* gene) [12]. Subsequently, both ends of lycopene are cyclized by PBS to produce β-carotene [11]. Finally, astaxanthin synthase (encoded by the *crtS* gene) introduces a keto group and a hydroxyl group to each of the β-carotene terminal rings to generate astaxanthin [13, 14]. Because astaxanthin synthase is a cytochrome P450 enzyme, it requires cytochrome P450 reductase, which is encoded by the *crtR* gene in *X. dendrorhous*, as an electron donor for its catalysis [15].

It is known that there is a relationship between the carbon source and the synthesis of carotenoids, but the molecular regulatory mechanisms involved in the *X. dendrorhous* carotenogenic pathway remain poorly understood. In cultures supplemented with glucose, carotenogenesis is induced only after the culture medium runs out of glucose, suggesting that carotenogenesis in *X. dendrorhous* is regulated by catabolic repression. Consistent with that hypothesis, the addition of glucose to cultures previously deprived of this sugar causes a complete inhibition of carotenoid synthesis and decreases in the transcript levels of the carotenogenic genes *crtI*, *crtYB* and *crtS* [16, 17]. Further, the promoter for *crtS* contains four consensus motifs for the binding of CreA [13], which is a negative regulator involved in catabolic repression in *Aspergillus nidulans* [18]. In addition, extracellular α-glucosidase and invertase activities were not detected in *X. dendrorhous* cultures when glucose was used as a carbon source, suggesting the catabolic repression of these enzymes [19, 20]. This indicates that this regulatory mechanism is operative in *X. dendrorhous*, as genes encoding glycosyl hydrolases are well known as targets of catabolic repression.

Catabolic repression has been widely studied in *Saccharomyces cerevisiae*, in which glucose has a repressive effect mainly at the transcriptional level for various groups of genes, with subsequent decreases in their expression products [21]. Glucose-repressed genes include those encoding proteins involved in the Krebs cycle, electron transport chain, glyoxylate cycle, uptake and metabolization of carbon sources alternative to glucose (such as the genes *GAL*, *SUC* and *MAL*) and gluconeogenesis. Additionally, some of the genes that encode high-affinity glucose transporters and many genes involved in the responses to various stresses are repressed by glucose [21].

In general, glucose repression in *S. cerevisiae* mainly depends on the subcellular localization of the regulator Mig1 (homologous to CreA in *A. nidulans*). At high glucose levels, the repressor Mig1 is dephosphorylated and localizes at the nucleus, where it recognizes and binds to specific regulatory sequences known as “Mig1 boxes” at the promoters of the target genes. Then, Mig1 recruits a co-repressor complex formed by Cyc8 and Tup1 that represses the transcription of the target genes. In the absence of glucose, Mig1 is phosphorylated by the Snf1 kinase complex, loses its interaction with the Cyc8–Tup1 complex, and is exported to the cytoplasm [22, 23]. In *S. cerevisiae*, the co-repressor complex Cyc8–Tup1 is considered a global transcriptional co-repressor because it regulates the expression of more than 180 genes, including those regulated by glucose. The proteins Cyc8 and Tup1 belong to highly evolutionarily conserved protein families, and similar repressors have been described in yeasts, worms, flies and mammals [24]. In *S. cerevisiae, CYC8* and/or *TUP1* knock-out mutations are not lethal but have pleiotropic effects causing diverse phenotypes, including slow growth, flocculation, sporulation and loss of certain aspects of glucose repression [24, 25]. Because the co-repressor complex does not bind directly to DNA, it is recruited to the different promoters by specific DNA-binding proteins. For example, genes repressed by glucose, induced by DNA damage or regulated by oxygen are recognized by the DNA-binding proteins Mig1, Crt1 and Rox1, respectively [24, 26].

Considering that catabolic repression is an important regulatory mechanism that is widely conserved among eukaryotes, and given that results from previous studies suggest that carotenogenesis in *X. dendrorhous* could be regulated by this mechanism, the aim of this work was to study the catabolic repression regulatory mechanism and carotenogenesis in *X. dendrorhous*, focusing on the role of the co-repressor complex Cyc8–Tup1.

## Results and discussion

### General characterization of catabolic repression in *X. dendrorhous*

**Fig. 2:**
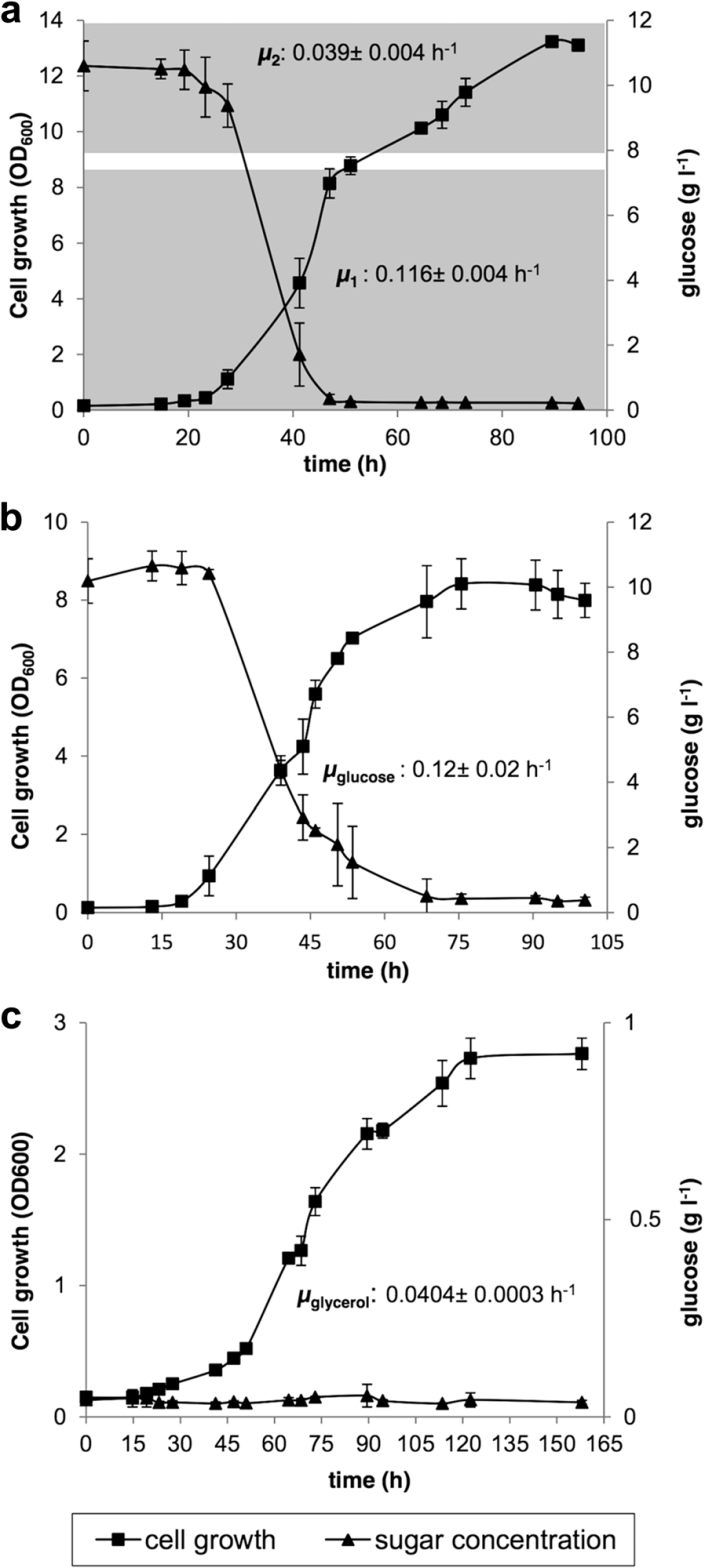
*Xanthophyllomyces dendrorhous* growth curves when cultured in MMv supplemented with glucose and/or glycerol. Growth curves at 22 °C using MMv supplemented with: **a** 1% glucose and 1% glycerol, **b** 1% glucose and **c** 1% glycerol. In **a**, **b** and **c**, the OD_600_ and remaining glucose in the culture medium were determined. µ: growth rate. Data are shown as the average values of three independent cultures, and the *error bars* represent the standard deviation

First, we studied whether the *X. dendrorhous* wild-type strain (UCD 67–385, ATCC 24230) demonstrates a functional catabolic repression mechanism in which the Cyc8 and Tup1 proteins would play an important role. According to descriptions of other yeasts, one consequence of catabolic repression is the preferred use of glucose over any alternative carbon sources, deferring their use until glucose has been completely consumed. The preferential use of glucose over an alternative carbon source can be evidenced by a characteristic growth rate change of the microbial culture, known as diauxic growth [27]. To assess whether *X. dendrorhous* preferentially uses glucose, restricting the use of a second carbon source until after the glucose has been consumed, the wild-type strain was cultured in Vogel minimum medium (MMv) supplemented with glucose (preferred carbon source) and/or glycerol (alternative carbon source) (Fig. 2). As previously reported [17, 28, 29], a diauxic-type growth curve was obtained when the yeast was cultivated with both carbon sources, indicating a change in the used carbon source with different growth rates (µ): µ_1_: 0.116 ± 0.004 and µ_2_: 0.039 ± 0.004 h^−1^. The two µ values correspond to those obtained by culturing the yeast with each carbon source independently: glucose (µ_glucose_: 0.12 ± 0.02 h^−1^) or glycerol (µ_glycerol_: 0.0404 ± 0.0003 h^−1^). Interestingly, the change in µ coincides with the time point at which glucose is exhausted from the culture medium, which occurs when the culture reaches an OD_600_ value of approximately 8. These results support a functional catabolic repression mechanism in *X. dendrorhous.*

### Identification of the *X. dendrorhousCYC8* and *TUP1* genes

**Fig. 3:**
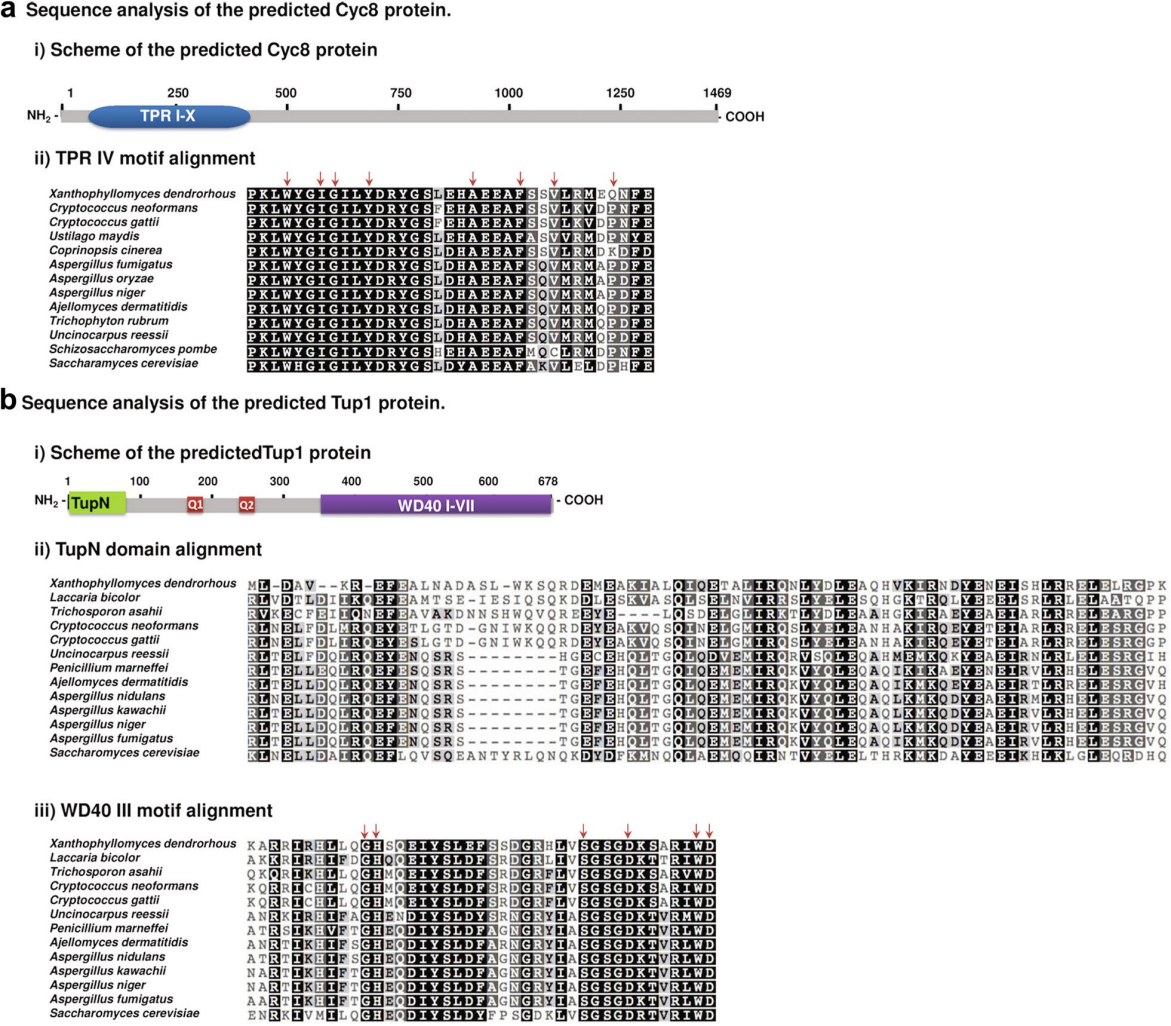
Sequence analysis of the *X. dendrorhous* Cyc8 and Tup1 predicted proteins. **a** Cyc8: (i) scheme of the deduced Cyc8 protein, in which ten TPR motifs (I–X) were identified; (ii) the alignment of TPR motif IV (residues 169 to 203 of the *X. dendrorhous* Cyc8 protein) is shown as an example. **b** Tup1: (i) scheme of the predicted Tup1 protein. Q1 and Q2 represent two glutamine-rich regions; (ii) sequence alignment of the identified TupN domain (residues 1 to 75 of the *X. dendrorhous* Tup1 protein); (iii) sequence alignment of the identified WD40 motif III (residues 430 to 470 of the *X. dendrorhous* Tup1 protein) is shown as an example. Alignments are colored by identity percentage as follows: *black* 100% identity, *grey* > 80% identity, *light grey* > 60% identity and *white* < 60% identity. Additionally, *red arrows* above some residues indicate residues required for the formation of the secondary structures of the TPR (a helix-turn-helix (HTH) structure) and WD40 (four antiparallel β sheets) motifs. For TPR alignment, the Cyc8 protein sequences of *X. dendrorhous* [KX517902], *C. neoformans* [XP_567714.1], *C. gattii* [KIR43866.1], *U. maydis* [XP_011391826.1], *C. cinerea* [XP_001828491.2], *A. fumigatus* [XP_755516.1], *A. oryzae* [XP_001821909.1], *A. niger* [XP_001399530.1], *A. dermatitidis* [EEQ 87432.1], *T. rubrum* [XP_003236691.1], *U. reesii* [XP_002583327.1], *S. pombe* [NP_596609.1], and *S. cerevisiae* [AJQ13886.1] were used. For TupN and WD40 alignments of the Tup1 protein, Tup1 sequences of *X. dendrorhous* [KX517903], *L. bicolor* [XP_001881168.1], *T. asahii* [EJT51849.1], *C. neoformans* [AFR95667.1], *C. gattii* [XP_003194397.1], *U. reesii* [XP_002543629.1], *P. marneffei* [AAL99251.1], *A. dermatitidis* [XP_002620234.1], *A. nidulans* [CBF70872.1], *A. kawachii* [GAA92251.1], *A. niger* [XP_001396553.2], *A. fumigatus* [XP_747562.1] and *S. cerevisiae* [EGA87747.1] were used. The GenBank or NCBI reference sequence is indicated in* brackets*

To identify the *X. dendrorhous CYC8* and *TUP1* genes, genomic sequences of the wild-type strain [30] were analyzed using the BLASTx tool from NCBI for comparisons with homologous sequences available in the GenBank database. Genomic regions of approximately 6.5 and 3.5 kb containing the potential *X. dendrorhous CYC8* and *TUP1* genes, respectively, were identified and uploaded to GenBank [KX517902] and [KX517903]. Additionally, transcriptomic sequences of the yeast were analyzed to identify the putative ORFs encoding both proteins, and by comparing the genomic and transcriptomic sequences, the structures of the potential *X. dendrorhous CYC8* and *TUP1* genes were determined. The *CYC8* gene has 9 introns and 10 exons with an ORF of 4410 bp, which would give a Cyc8 protein of 1469 amino acids (Fig. 3a). BLASTp analysis of the amino acid sequence revealed high identity with Cyc8 proteins from other microorganisms. The search for conserved domains identified 10 tetratricopeptide repeats (TPRs) in the N-terminal region of the protein; these motifs are essential for the structure and function of Cyc8, allowing the interaction with Tup1 and with several DNA-binding regulators, such as Mig1 [24]. Although various types of TPRs have been described in relation to the consensus sequence, the motif found in the *X. dendrorhous* Cyc8 protein coincides in sequence and position with TPR motifs found in Cyc8 proteins described in other organisms, including *S. cerevisiae* [24, 31, 32], suggesting structural similarities because these tandem TPRs usually fold into a helix-turn-helix (HTH) structure [33]. Notably, the *X. dendrorhous* Cyc8 protein would be considerably larger than others described in other yeasts, such as *S. cerevisiae* (966 amino acid residues) [34], with approximately five hundred additional amino acids at the C-terminus. However, several studies have indicated that the N-terminal domain, which has the TPR repeats in the *S. cerevisiae* Cyc8 protein and equivalent proteins in other organisms, is the only functional domain of this co-repressor, and this domain is present in the deduced Cyc8 protein from *X. dendrorhous.*

The *TUP1* gene is composed of 10 introns and 11 exons. An ORF of 2037 bp was identified, and its in silico translation gave a protein of 678 residues (Fig. 3b). In the N-terminal region of the *X. dendrorhous* Tup1 protein, the characteristic TupN domain necessary for Tup1 oligomerization [35] and for the interaction with Cyc8 [36] was identified. Additionally, two glutamine-rich regions characteristic of Tup1 proteins in other organisms were identified. The C-terminal region was identified as containing the functional domain, which is composed of seven WD40 repeats with a characteristic secondary structure rich in β-sheets (four antiparallel β-sheets in each WD40 motif) and which is essential for Tup1 function as it is required for protein–protein interactions with Cyc8 and other proteins [36]. The sequence alignment with Tup1 proteins from other microorganisms shows that the *X. dendrorhous* Tup1 protein contains the amino acids required for the structure and function of this protein. These analyses suggest that the *X. dendrorhous* genes identified as *CYC8* and *TUP1* encode Cyc8 and Tup1 proteins that are orthologs of those described in *S. cerevisiae* and in other microorganisms.

### *Xanthophyllomyces dendrorhousCYC8* and *TUP1* genes heterologous complementation in *S. cerevisiae* mutant strains

To functionally characterize the *X. dendrorhous CYC8* and *TUP1* genes, heterologous complementation assays were performed using *cyc8* ^−^ and *tup1* ^−^ *S. cerevisiae* mutant strains (strains *Sccyc8* ^−^ and *Sctup1* ^−^, respectively). The expression vectors YEpNP–CYC8 and YEpNP–TUP1 carrying the respective *X. dendrorhous* cDNA were obtained, sequenced and used to transform the corresponding *S. cerevisiae* mutant strains. The transformants carrying the respective expression vectors and constitutively expressing the *X. dendrorhous CYC8* and *TUP1* genes were named *Sccyc8* ^−^ + YEpNP–CYC8 and *Sctup1* ^−^ + YEpNP–TUP1, respectively. Control strains containing the empty vector were obtained and named *Sccyc8* ^−^ + YEpNP and *Sctup1* ^−^ + YEpNP (Table 1).

**Table 1:** Strains and plasmids used in this work

Strain/plasmid	Description	Reference or source
Strains		
*E. coli*		
DH5α	Amp^S^	[37]
*X. dendrorhous*		
UCD 67–385	Diploid wild-type strain (Hyg^S^ and Zeo^S^)	[3]; ATCC 24230
385*cyc8* ^−^	Hyg^R^ and Zeo^R^ homozygous mutant (*cyc8* ^−^/*cyc8* ^−^ *)* that derives from UCD 67–385. A *CYC8* allele was interrupted by a module that confers resistance to hygromycin B and the other allele by a module that confers resistance to zeocin	This work
385*tup1* ^−^	Hyg^R^ and Zeo^R^ homozygous mutant (*tup1* ^−^/*tup1* ^−^ *)* that derives from UCD 67–385. A *TUP1* allele was interrupted by a module that confers resistance to hygromycin B and the other allele by a module that confers resistance to zeocin	This work
*S. cerevisiae*		
S288c	Haploid reference strain	ATCC 204508
YBR112C BY4741 (*Sccyc8* ^−^)	Haploid *cyc8* ^−^ mutant strain derived from S288c. Genotype: *MATa his3delta1 leu2delta0 met15delta0 ura3delta0 deltaCYC8*	[38]; ATCC 4007161
*Sccyc8* ^−^+YEpNP-CYC8	*Sccyc8* ^−^ strain carrying YEpNP-CYC8 (vector to express the *X. dendrorhous CYC8* gene)	This work
*Sccyc8* ^−^+YEpNP	*Sccyc8* ^−^ strain carrying YEpNP (empty vector)	This work
YCR084C BY4741 (*Sctup1* ^−^)	Haploid *tup1* ^−^ mutant strain derived from S288c. Genotype: *MATa his3delta1 leu2delta0 met15delta0 ura3delta0 deltaTUP1*	[38]; ATCC 4007198
*Sctup1* ^−^+YEpNP-TUP1	*Sctup1* ^−^ strain carrying YEpNP-TUP1 (vector to express the *X. dendrorhous TUP1* gene)	This work
*Sctup1* ^−^ + YEpNP	*Sctup1* ^−^ strain carrying YEpNP (empty vector)	This work
Plasmids		
pBluescript II SK (−) XR	Cloning vector. (AmpR, pUC ori, white/blue colony selection)	Stratagene, La Jolla, CA, USA
pMN-*hph*	pBluescript II SK (−) XR containing at the *Eco*RV site a 1.8 kb module that confers resistance to hygromycin B	[39]
pIR-*zeo*	pBluescript II SK (−) XR containing at the *Eco*RV site a 1.2 kb module that confers resistance to zeocin	[7]
pgCYC8	pBluescript II SK (−) XR containing at the *Eco*RV site a 2.4 kb *X. dendrorhous* genomic fragment of the *CYC8* gene	This work
pgCYC8-Hyg	pgCYC8 containing at the *Bmg*BI site the hygromycin B resistance module disrupting the *CYC8* gene fragment	This work
pgCYC8-Zeo	pgCYC8 containing between *Pml*I and *Bmg*BI sites the zeocin resistance module replacing 527 bp of the *CYC8* gene fragment	This work
pgTUP1	pBluescript II SK(−)XR containing at *Eco*RV site a 2.7 kb *X. dendrorhous* genomic fragment of the *TUP1* gene	This work
pgTUP1-Hyg	pgTUP1 containing the hygromycin B resistance module replacing a 2.0 kb *Eco*RV fragment inside the *TUP1* gene fragment	This work
pgTUP1-Zeo	pgTUP1 containing the zeocin resistance module replacing a 2.0 kb *Eco*RV fragment inside the *TUP1* gene fragment	This work
YEpNP	*S. cerevisiae* expression vector modified from YEpAct4 [40]. *LEU2* gene auxotrophy marker. It has a *Xba*I restriction site between the Act4 constitutive promoter and the TDH3t terminator	[41]
YEpNP-CYC8	*S. cerevisiae e*xpression vector derived from YEp-NP containing at the *Xba*I site between the Act4 promoter and the TDH3t terminator the *X. dendrorhous CYC8* cDNA	This work
YEpNP-TUP1	*S. cerevisiae e*xpression vector derived from YEp-NP containing at the *Xba*I site between the Act4 promoter and the TDH3t terminator the *X. dendrorhous TUP1* cDNA	This work

*Amp* ^*S*^ sensitive to ampicillin, *Hyg* ^*S*^ sensitive to hygromycin B, *Hyg* ^*R*^ resistant to hygromycin B, *Zeo* ^*S*^ sensitive to zeocin, *Zeo* ^*R*^ resistant to zeocin, *ATCC* American Type Culture Collection, *AmpR* ampicillin resistance, *ColE1 ori* replication origin of *E. coli* ColE1 plasmid

**Fig. 4:**
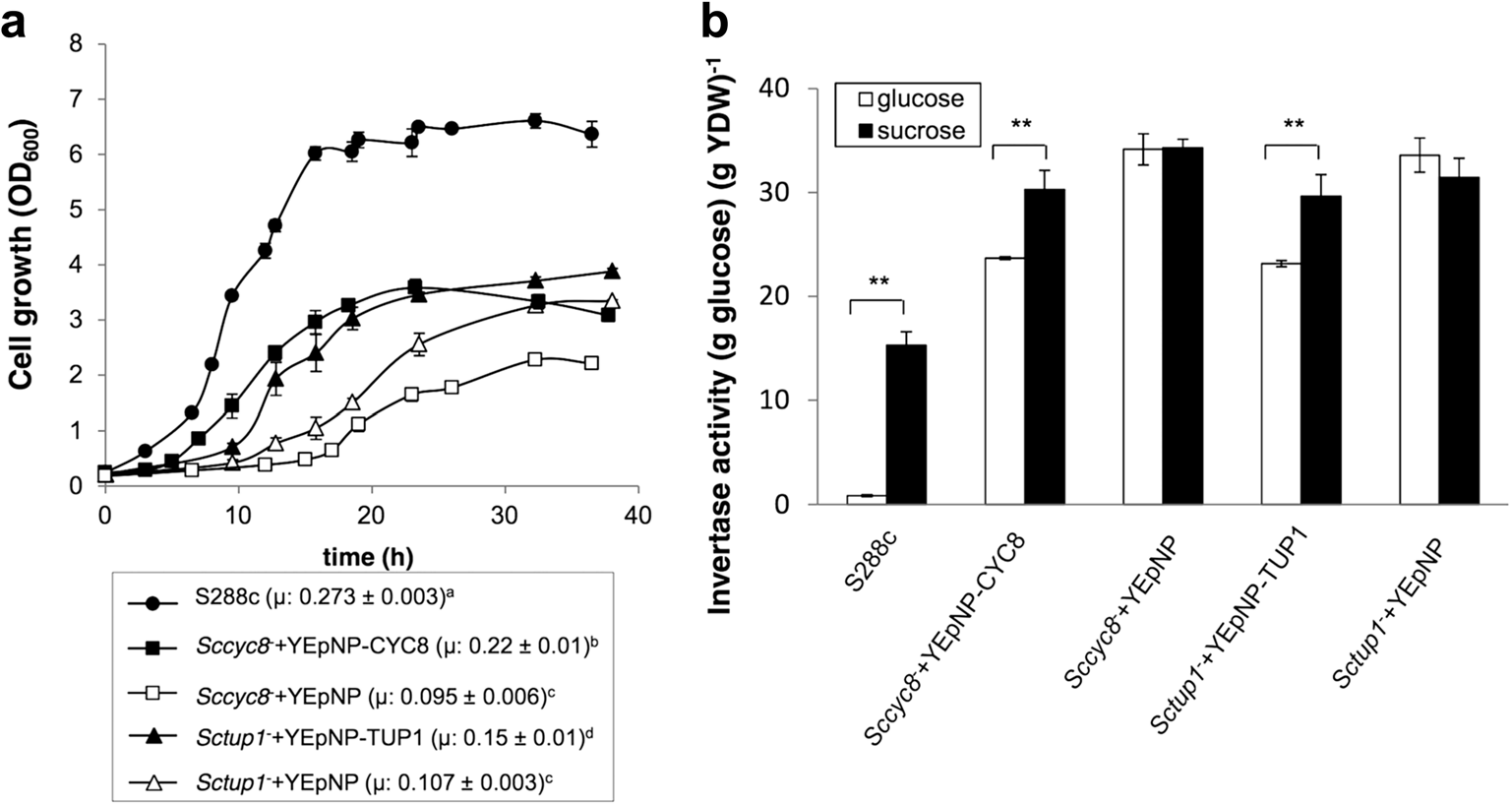
Heterologous complementation of the *X. dendrorhous CYC8* and *TUP1* genes in *S. cerevisiae*. **a** Growth curves of strains S288c, Sc*cyc8* ^−^+YEpNP-CYC8, Sc*cyc8* ^−^+YEpNP, Sc*tup1* ^−^+YEpNP-TUP1 and Sc*tup1* ^−^+YEpNP cultured in SD-2% glucose at 30 °C. Significant differences between µ values are represented by different letters (*a*–*d*) based on ANOVA with Tukey’s post-test (*α* = 0.05; *p* ≤ 0.05). **b** Invertase activity in samples taken from cultures at the early exponential growth phase. The strains were cultured at 22 °C in SD-2% glucose (repressive conditions) or SD-2% sucrose (non-repressive conditions). *YDW* yeast dry weight. The data represent the average of three independent cultures with their respective standard deviations (*error bars*). **p < 0.01, Student’s t test

In *S. cerevisiae*, the *tup1* ^−^ and *cyc8* ^−^ mutations have pleiotropic effects, including growth delay and loss of some aspects of catabolic repression. For example, these mutations repress the use of carbon sources other than glucose, including by repressing the expression of the *SUC2* gene, which encodes β-fructofuranosidase (invertase), an enzyme required for using sucrose as a carbon source [24, 25, 42]. Thus, yeast growth, the use of sucrose as an alternative carbon source under repressive conditions, and invertase activity were evaluated in the complemented and control strains. Thus, growth curves from strains S288c (reference strain), *Sccyc8* ^−^ + YEpNP–CYC8, *Sctup1* ^−^ + YEpNP–TUP1 and controls were obtained using glucose as the sole carbon source (Fig. 4a). Strains *Sccyc8* ^−^ + YEpNP–CYC8 and *Sctup1* ^−^ + YEpNP–TUP1 had µ values of 0.22 ± 0.01 and 0.15 ± 0.01 h^−1^, respectively, corresponding to generation times (gt) of 3.1 ± 0.2 and 4.7 ± 0.4 h. Notably, the growth rates of strains expressing the *X. dendrorhous CYC8* and *TUP1* genes were approximately 2.4- and 1.5-fold higher than the respective control strains carrying the YEpNP empty vector (μ: 0.095 ± 0.006 and 0.107 ± 0.003 h^−1^, respectively). Nevertheless, the wild-type strain had a μ value of 0.273 ± 0.003 h^−1^ (gt: 2.52 ± 0.03 h), which is the highest μ of all the strains evaluated. Considering that the optimal growth temperature of the *X. dendrorhous* wild-type strain is 22 °C and that these *S. cerevisiae* strains were expressing *X. dendrorhous* genes, growth was also evaluated at 22 °C. Consistent with the previous observation, the *Sccyc8* ^−^ + YEpNP and *Sctup1* ^−^ + YEpNP control strains showed the lowest µ values (0.069 ± 0.001 and 0.071 ± 0.001 h^−1^, respectively), and the wild-type strain had the highest growth rate (0.159 ± 0.003 h^−1^), while *Sccyc8* ^−^ + YEpNP–CYC8 and *Sctup1* ^−^ + YEpNP-TUP1 had intermediate growth rate values of 0.094 ± 0.001 and 0.085 ± 0.001 h^−1^, respectively. These results indicate that *S. cerevisiae* strains carrying *X. dendrorhous CYC8* or *TUP1* show an intermediate growth phenotype between the reference strain and the corresponding control strains bearing empty vectors, suggesting a partial complementation of the *S. cerevisiae cyc8* ^−^ and *tup1* ^−^ mutations by the corresponding *X. dendrorhous* homologous genes. Considering that cultures at 22  and 30 °C showed similar growth profiles and that the genes under investigation derive from *X. dendrorhous*, cultures for the following heterologous complementation analyses were performed at 22 °C.

The effect of glucose on the extracellular invertase activity in the complemented *S. cerevisiae* strains was determined. For this purpose, the five strains S288c, *Sccyc8* ^−^ + YEpNP–CYC8, *Sctup1* ^−^ + YEpNP–TUP1, *Sccyc8* ^−^ + YEpNP and *Sctup1* ^−^ + YEpNP were cultured in SD medium supplemented with either glucose (repressive conditions) or sucrose (non-repressive conditions) (Fig. 4b). Extracellular invertase activity was determined at the early exponential phase of growth, when the concentration of glucose in the medium was still high (approximately 15 g l^−1^), and at the stationary phase of growth, when the concentration of the remaining glucose in the medium was less than 3 g l^−1^. Unlike the control strains carrying empty vectors, in strain S288c, the extracellular invertase activity at the early exponential phase of growth was repressed in the presence of glucose but not in the presence of sucrose (Fig. 4b). For the complemented strains cultivated with glucose, the extracellular invertase activity was approximately 30% lower than that in the respective control strains (Fig. 4b). Additionally, in cultures of the complemented strains using glucose or sucrose as the sole carbon source, the extracellular invertase activity was approximately 25% lower for strains expressing *X. dendrorhous* genes and cultured with glucose than for the same strains cultured with sucrose. No statistically significant differences were observed between samples from these two conditions in the control strains carrying the empty vector (Fig. 4b). Finally, as expected, there were no statistically significant differences between the invertase activities of samples from cultures supplemented either with glucose or sucrose taken at the stationary phase of growth. This is likely because at this growth phase, the glucose in the medium has been largely consumed, eliminating the glucose-dependent repression. Together, these results also indicate that the *X. dendrorhous CYC8* and *TUP1* genes partially complement the *S. cerevisiae cyc8* ^−^ and *tup1* ^−^ mutations in the glucose-dependent repression of extracellular invertase activity because an intermediary phenotype between strain S288c and the control mutant strains was observed, indicating that both *X. dendrorhous* genes are functional in the *S. cerevisiae* catabolic repression mechanism.

Furthermore, one consequence of catabolic repression is the repression of genes involved in the utilization of carbon sources other than glucose, such as sucrose. Thus, a yeast with a functional catabolic repression mechanism would not be able to use a carbon source other than glucose in the presence of the non-hydrolysable glucose analog 2-deoxy-d-glucose (2DG). To evaluate this scenario, the five *S. cerevisiae* strains were cultured in SD minimal medium supplemented with sucrose with or without 2DG (Table 2). The µ value of strain S288c cultured with only sucrose (control condition) was 2.2 times that of the same strain cultured in the presence of sucrose and 2DG (repressive conditions), suggesting that the machinery required for using sucrose as a carbon source is not expressed in the latter condition. This is also mirrored in the maximum OD_600_ that was reached after 65 h of culture, which was approximately threefold higher in the control condition. Additionally, the *Sccyc8* ^−^ + YEpNP–CYC8 and *Sctup1* ^−^ + YEpNP–TUP1 strains showed a slower growth rate when cultured in the presence of 2DG compared to the control condition (sucrose only), reaching μ values approximately 1.3 and 1.2 times lower, respectively. Moreover, the maximum OD_600_ values reached by the *Sccyc8* ^−^ + YEpNP–CYC8 and *Sctup1* ^−^ + YEpNP–TUP1 strains cultured with only sucrose were 1.4- and 1.3-fold higher than those for the same strains grown in the presence of both sucrose and 2DG, respectively. Finally, the control strains *Sccyc8* ^−^ + YEpNP and *Sctup1* ^−^ + YEpNP did not show differences between the two culture conditions in either their μ value or their maximum OD_600_. These results further support the hypothesis that the *X. dendrorhous CYC8* and *TUP1* gene products are functional in *S. cerevisiae* and complement the respective mutation by repressing the ability to use sucrose as a carbon source in the presence of 2DG. This result provides further evidence that these *X. dendrorhous* genes encode functional proteins that are involved in catabolic repression.

**Table 2:** Growth parameters from *S. cerevisiae* strains cultured with sucrose with or without 2-deoxy-d-glucose

Strain	µ_suc_ (h^−1^)	µ_suc-2DG_ (h^−1^)	µ_suc_/µ _suc-2DG_	OD_max suc_	OD_max suc-2DG_	OD_max suc_/OD_max suc-2DG_
S288c	0.165 ± 0.002	0.074 ± 0.002**	2.23	7.2 ± 0.2	2.4 ± 0.1**	3.0
*Sccyc8* ^−^ + YEpNP-CYC8	0.105 ± 0.003	0.082 ± 0.003**	1.28	5.2 ± 0.2	3.73 ± 0.03**	1.39
Sc cyc8^−^ + YEpNP	0.068 ± 0.001	0.072 ± 0.001	0.94	2.7 ± 0.2	2.5 ± 0.3	1.08
*Sctup1* ^−^ + YEpNP-TUP1	0.095 ± 0.001	0.079 ± 0.002**	1.20	4.0 ± 0.2	3.16 ± 0.06**	1.27
Sc tup1^−^ + YEpNP	0.077 ± 0.005	0.069 ± 0.001	1.10	3.57 ± 0.07	3.5 ± 0.2	1.02

Strains were cultured at 22 °C in SD medium supplemented only with 2% sucrose (suc) or that in addition to 0.02% 2-deoxy-d-glucose (suc-2DG). Each µ value corresponds to the average of three independents cultures and errors to the standard deviation. OD_max_: maximum value of OD_600_ reached during the time period studied

** p < 0.01, Student’s t test (compared to the control condition)

For all of the analyzed parameters (growth and use of alternative to glucose carbon sources), the complemented *S. cerevisiae* strains harboring the corresponding *X. dendrorhous* genes presented an intermediate phenotype (between the reference and the control strains). Thus, it is noteworthy that in previous complementation studies of *CYC8* and *TUP1* homologs from other organisms in *S. cerevisiae,* it has been consistently observed that even when strains are complemented with the endogenous genes using either multi-copy or single-copy expression vectors, a full complementation of the respective mutations is not observed when using the repression of extracellular invertase activity and flocculation as criteria [43]. It is therefore not surprising that the genes isolated from *X. dendrorhous* only partially complemented the *cyc8* ^−^ and *tup1* ^−^ mutations in *S. cerevisiae*. Furthermore, Cyc8 and Tup1 must establish protein–protein interactions with each other and with DNA-binding regulators to perform their functions; thus, it is possible that the interactions between the *X. dendrorhous* proteins and their *S. cerevisiae* Cyc8–Tup1 complex counterparts or with Mig1 are not as strong or stable as with the orthologous endogenous protein.

### Phenotypic analyses of *cyc8*^−^ and *tup1*^−^*X. dendrorhous* mutant strains

To assess the functionality of the *TUP1* and *CYC8* genes in *X. dendrorhous*, *cyc8* ^−^ and *tup1* ^−^ mutant strains deriving from the diploid UCD 67–385 (385) wild-type strain [3] were obtained by gene replacement mutagenesis through homologous recombination [39]. The homozygous *cyc8* ^−^ mutant strain 385*cyc8* ^−^ was obtained after the sequential transformation of the wild-type strain using the insert DNA from plasmids pgCYC8-Hyg and pgCYC8-Zeo as transformant DNA. Meanwhile, the homozygous *tup1* ^−^ mutant 385*tup1* ^−^ was obtained by transforming the wild-type strain with the insert DNA from plasmid pgTUP1-Zeo and then of plasmid pgTUP1-Hyg. The corresponding mutations in the obtained mutant strains were confirmed by PCR analysis using comprehensive sets of primers (Additional file 1: Table S1) that allowed us to evaluate each locus using the corresponding gDNA as a template. It should be noted that the deleted regions of both genes encode elements that are essential for protein function; in the case of *CYC8*, the mutated region included the coding sequence of the N-terminal region containing the TPR motifs of the Cyc8 protein, and in the case of *TUP1*, it included the coding sequence of the C-terminal region bearing the functional domain containing the WD40 structural motifs. Several studies have shown that yeasts are particularly sensitive to various types of mutations compromising the named regions, causing loss of the functions of the respective proteins. Consistent with this, it has been observed that a *S. cerevisiae* mutant variant of the Tup1 protein lacking the C-terminus causes the same phenotype as the gene knockout mutation, while in the case of Cyc8, the expression of only the N-terminal domain of the protein is necessary and sufficient to obtain a wild-type phenotype [43, 44]. Considering that Cyc8 and Tup1 are general co-repressors of transcription that work together with different proteins to regulate the expression of various genes, it is expected that mutations of the corresponding *X. dendrorhous* genes would have pleiotropic effects affecting various aspects of cellular function such as invertase activity, growth and carotenogenesis.

**Fig. 5:**
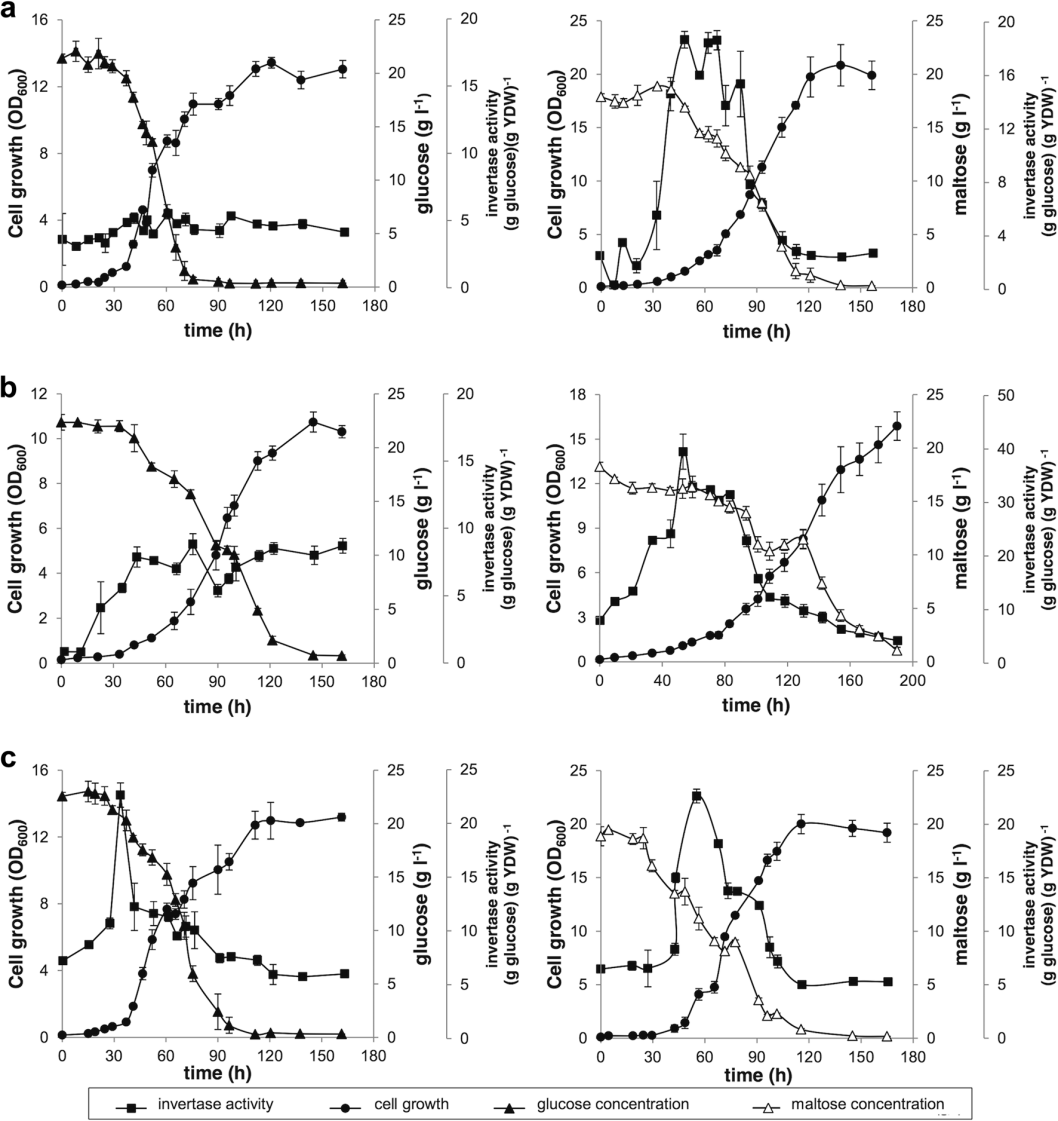
Growth curves and extracellular invertase activity in the wild-type UCD 67–385, 385*cyc8* ^−^ and 385*tup1* ^−^ strains. Data from strains **a** wild-type, **b** 385*cyc8* ^−^ and **c** 385*tup1* ^−^. Growth curves of the strains cultured in MMv-2% glucose (*left panels*) or MMv-2% maltose (*right panels*) at 22 °C. The extracellular invertase activity and glucose or maltose concentration were measured over time. *YDW* yeast dry weight. The average value of three independent cultures is shown, and the *error bars* represent the standard deviation

Once the 385*cyc8* ^−^ and 385*tup1* ^−^ strains were constructed, the effects of these mutations on the glucose-mediated repression of the extracellular invertase activity in *X. dendrorhous* were evaluated. The wild-type, 385*cyc8* ^−^ and 385*tup1* ^−^ strains were cultured in MMv supplemented with maltose (non-repressive conditions) or with glucose (repressive conditions), and the extracellular invertase activity was measured along the growth curve (Fig. 5). Under repressive conditions, the mutant strains had a higher extracellular invertase activity than did the wild-type strain along the growth curve, with strain 385*tup1* ^−^ having the highest activity. Additionally, strains 385*cyc8* ^−^ and 385*tup1* ^−^ reached higher enzymatic activity values than those of the wild-type, approximately 2- and 4- fold higher, respectively, when cultured with glucose. In contrast, the three strains showed similar activity patterns when cultured under non-repressive conditions. These results indicate that in both *X. dendrorhous* mutant strains, the mechanism for repressing extracellular invertase activity was impaired compared to that of the wild-type, as expected based on the *S. cerevisiae* heterologous complementation results.

**Fig. 6:**
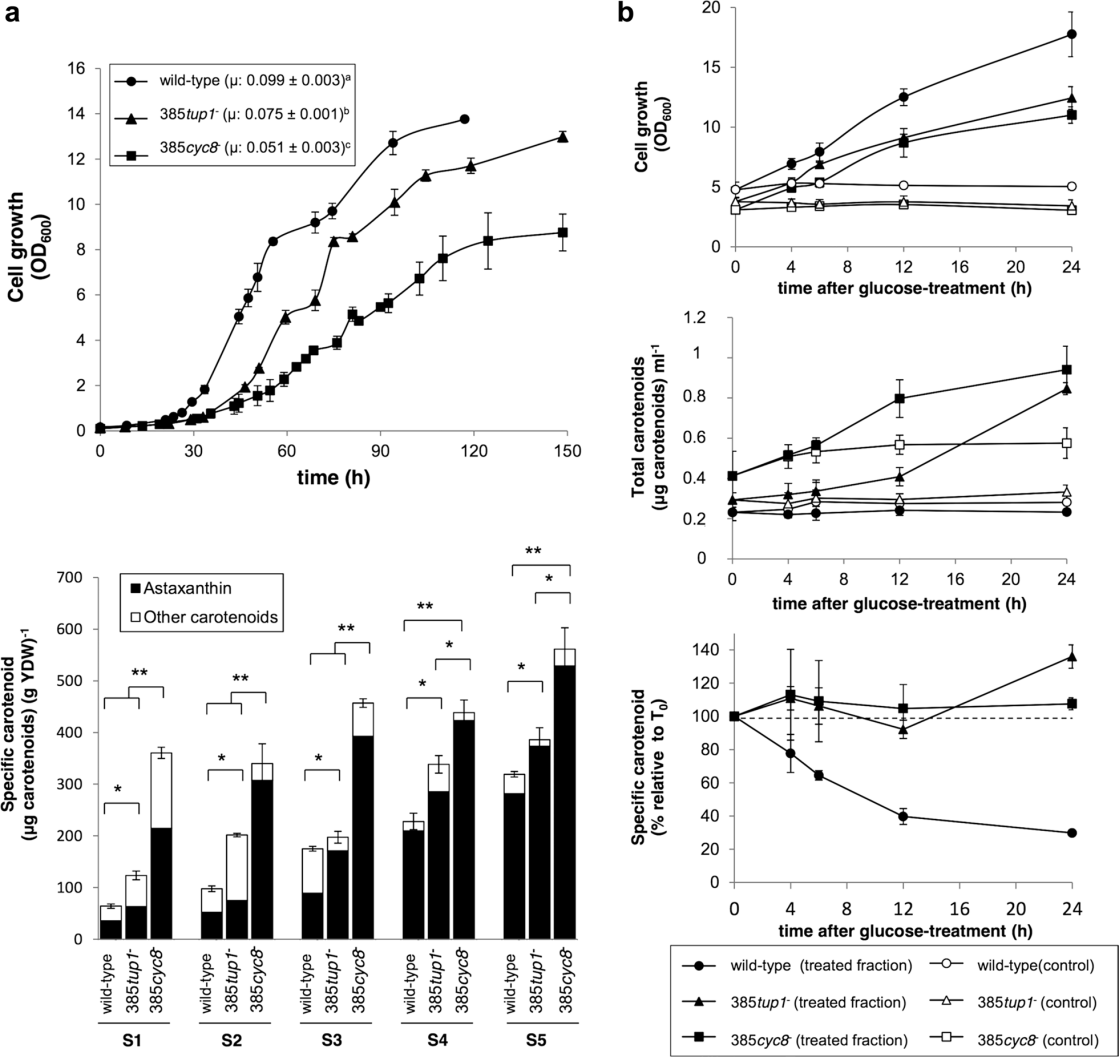
Growth curves, carotenoid production and the effect of glucose on carotenoid production. Data from the wild-type, 385*cyc8* ^−^ and 385*tup1* ^−^ strains are shown. **a** Strains were cultured at 22 °C in MMv with 2% glucose. *Top* growth curves. *Bottom* specific carotenoid production at the S1, S2, S3, S4 and S5 growth phases (described in the text). The contents of astaxanthin and of other carotenoids (including neurosporene, γ-carotene, β-carotene, echinenone, hydroxy-echinenone and phoenicoxanthin) in each sample are represented in* black* and* white*, respectively. **b** *Top* growth curves. *Middle* total carotenoids produced per volume of culture during 24 h after glucose treatment. *Bottom* specific carotenoid production [(µg of carotenoid) (g yeast dry weight)^−1^] relative to the values before treatment (T_0_). *YDW* yeast dry weight. In all cases, values correspond to the average of three independent cultures, and *error bars* represent the standard deviation. *p < 0.05 and **p < 0.01, Student’s t test. Significant differences between µ values, according to ANOVA and Tukey’s post-test (α = 0.05; p ≤ 0.05), are represented by different letters (*a*–*c*)

The effects of the *cyc8* ^−^ and *tup1* ^−^ mutations in *X. dendrorhous* on growth were also evaluated (Fig. 6a). The calculated µ values were 0.099 ± 0.003, 0.051 ± 0.003 and 0.075 ± 0.001 h^−1^ for strains wild-type, 385*cyc8* ^−^ and 385*tup1* ^−^, respectively, confirming that the mutant strains showed slower growth than the wild-type, as in *S. cerevisiae*. Additionally, cultures of the mutant strain 385*cyc8* ^−^ reached the lowe1st OD_600_ value at the stationary growth phase.

It should be mentioned that while glucose-dependent repression of extracellular invertase activity is a well-known example of catabolic repression, to the best of our knowledge, the regulation of carotenogenesis by catabolic repression has been suggested only recently [29]. Considering previous experimental evidence indicating that glucose represses the synthesis of carotenoids in *X. dendrorhous* [16, 17], the effects of the *cyc8* ^−^ and *tup1* ^−^ mutations on carotenogenesis were also evaluated. Samples were collected at 5 different time points during the growth curve, representing 5 different growth phases: (i) S1: early exponential (OD_600_: 1–2), (ii) S2: exponential (OD_600_: 4–5), (iii) S3: late exponential (OD_600_: 6–7), (iv) S4: early stationary (OD_600_: 8–10), and (v) S5: late stationary (OD_600_: 9–12, depending on the strain). These samples were used for extraction, quantification and analysis of the carotenoids produced by each strain (Fig. 6a). The specific carotenoid production [(µg of carotenoids) (g of dry yeast)^−1^] increased in both mutant strains at all time points analyzed, reaching production levels at the stationary growth phase that were approximately 90 and 40% higher in strains 385*cyc8* ^−^ and 385*tup1* ^−^, respectively, compared to the wild-type. These observations suggest that the glucose-mediated repression of carotenogenesis requires both functional co-repressors (Cyc8 and Tup1). In addition, the astaxanthin fraction in the mutant strains 385*cyc8* ^−^ and 385*tup1* ^−^ was also greater than in the wild-type (80–85 vs 70%) (Fig. 6a). The detailed analysis of carotenoid composition of each sample is included as supplementary material (Additional file 2: Table S2, Additional file 3: Figure S1).

Previous studies have demonstrated that the addition of glucose to the culture medium causes total inhibition of carotenoid production in the *X. dendrorhous* wild-type strain [16]. Moreover, strains 385*cyc8* ^−^ and 385*tup1* ^−^ showed higher carotenoid production from the beginning of the culture (when the glucose concentration in the cultures was high), suggesting that carotenoid production is not regulated by glucose in these strains. Based on the above and to determine whether these mutations override the repressive effect of glucose on carotenogenesis, the effect of glucose on carotenoid production was assessed in the strains wild-type, 385*cyc8* ^−^ and 385*tup1* ^−^. Carotenoid production during a short period after glucose addition was evaluated as previously described [16]. The strains were cultured in YM medium at 22 °C without glucose supplementation for up to 24 h after the stationary phase of growth was reached; at this point, each culture was divided into two fractions. One fraction was supplemented with 2% glucose, and the other was left untreated as a control. Each fraction was sampled during 24 h, and carotenoid production was analyzed (Fig. 6). The addition of glucose increased the biomass in cultures from all the strains, evidenced as an increased OD_600_. In contrast, the amount of carotenoids produced per ml of culture did not change even 24 h post-treatment in the wild-type cultures after glucose addition, whereas under the same conditions, carotenoid production was observed in the mutant 385*cyc8* ^−^ and 385*tup1* ^−^ cultures, with carotenoid levels at 24 h post-treatment being approximately 2- and 2.5-fold, respectively, the amounts of carotenoids produced in these strains before treatment (Fig. 6b). The specific amount of carotenoids (carotenoids per biomass unit) showed a progressive decrease over time after glucose addition in the wild-type strain cultures, becoming approximately 70% lower than the value before treatment (Fig. 6c). This indicates that the addition of glucose to the wild-type strain cultures favors growth but that there is no carotenoid synthesis. In contrast, the addition of glucose to cultures of the mutant strains 385*cyc8* ^−^ and 385*tup1* ^−^ did not affect the amount of carotenoids per biomass unit during the timespan analyzed (Fig. 6c), indicating that the biomass increment is accompanied by carotenoid production, which does not occur in the wild-type strain. These results show that the cyc*8* ^−^ and *tup1* ^−^ mutations in *X. dendrorhous* avoid the glucose-mediated repression of carotenogenesis in this yeast, indicating that these genes are involved in regulating carotenogenesis.

To complement the carotenoid production analysis and considering that the co-repressors Cyc8 and Tup1 would be acting at the transcriptional level, the expression levels of several genes involved in carotenoid synthesis were evaluated by RT-qPCR (Additional file 4: Figure S2) during a short time period after the addition of glucose, as previously described [16]. The results showed glucose-dependent differences between the mutant strains and the wild-type yeast in the transcript levels of some of the genes studied. The greatest differences were observed during the first 6 h post-treatment, when glucose is still elevated in the culture medium (approximately 15 g l^−1^).

The genes *INV* and *grg2* were used as controls because they are known targets of glucose repression [16, 29]. As expected, both genes showed lower transcript levels in the glucose-treated fraction compared to the control in the wild-type strain (approximately 12- and 600- fold for *INV* and *grg2*, respectively). However, in the 385*cyc8* ^−^ and 385*tup1* ^−^ strains, the *INV* repression level was clearly lower (approximately fivefold in both mutant strains). Additionally, an increase in the *INV* transcript levels was observed 6 h post-treatment in 385*cyc8* ^−^ strain, probably due to a relief of the transcriptional repression of the *INV* gene. In the case of the *grg2* gene, the most pronounced difference was observed in the strain 385*cyc8* ^−^, in which the repression level in the glucose-treated fraction was only 25-fold compared to the control. These results indicate that Cyc8 and Tup1 are effectively acting at the transcriptional level in *X. dendrorhous*. The effects of the *cyc8* ^−^ and *tup1* ^−^ mutations were also analyzed at the transcriptional level of genes involved in carotenogenesis. Greater effects were observed for genes involved in the synthesis of carotenoid precursors, including *HMGR*, *idi* and *FPS.* The *HMGR* gene encodes the enzyme 3-hydroxy-3-methylglutaryl-CoA reductase, which catalyzes the synthesis of mevalonate from which IPP (the general precursor of isoprenoids) is synthesized [2, 7]. In the strain 385*cyc8* ^−^, the *HMGR* transcript levels after the addition of glucose were higher than in the control fraction, but this effect was not observed in the 385*tup1* ^−^ mutant or the wild-type strain. This observation also supports the difference in carotenoid production observed between the two mutant strains, given that previous studies indicate that increasing the available mevalonate leads to an increase in carotenoid production in *X. dendrorhous* [2]. In the case of the *idi* gene, the mRNA level was affected in both mutant strains, showing an increase after glucose addition of 5- to 15-fold, which was not observed in the wild-type strain. Additionally, in 385*cyc8* ^−^ strain, an eightfold increase in the *FPS* transcript level was observed after glucose addition, while in 385*tup1* ^−^ and wild-type strains, this increase was approximately 3- to fourfold. The higher *FPS* transcript levels may also explain the increased carotenoid production in strain 385*cyc8* ^−^, as the overexpression of this gene in *X. dendrorhous* has been shown to lead to higher carotenoid production [10].

Repression of the *crtE*, *crtI* and *crtYB* carotenogenic genes was observed in both mutant strains and in the wild-type yeast in response to the addition of glucose. However, the repression of *crtE* is alleviated earlier in the mutant strains than in the wild-type strain. Furthermore, in the mutant strain 385*cyc8* ^−^, the repression of *crtI* (approximately twofold) is milder than that in the wild-type (approximately fivefold). The *crtI* transcript in the treated fraction from the 385*tup1* ^−^ strain reached levels even higher than in the control, which was not observed in the wild-type strain. A similar effect was observed for the *crtYB* gene: the *crtYB* gene transcript levels increased after repression in both mutant strains, which did not occur in the wild-type. In the case of the *crtS* gene, no differences were observed among the three strains. Finally, the addition of glucose to the culture medium increased the *crtR* transcript levels in all three strains, but the increase was smaller in the 385*cyc8* ^−^ strain than in the other two. Regarding the latter, it should be noted that in most yeasts, there is a single gene encoding cytochrome P450 reductase, *crtR* in the case of *X. dendrorhous*, and that the cytochrome P450 reductase acts as an electron donor for various cytochrome P450 monooxygenases [15]. Three genes encoding cytochrome P450 monooxygenases have been functionally described in *X. dendrorhous*, including *crtS*, which is involved in carotenogenesis, and *CYP51* [41] and *CYP61* [7], which are involved in ergosterol biosynthesis. The increased *crtR* transcript level may also favor the synthesis of compounds other than carotenoids that compete for carotenogenesis precursors, similar to the way ergosterol synthesis negatively affects carotenoid synthesis. This mechanism may also explain the difference observed between the mutant strains regarding carotenoid production. In summary, the differences observed between the wild-type and the mutant strains regarding the effects of glucose on the transcript levels of some genes involved in carotenogenesis can at least partly explain the increase in carotenoid production in the mutant strains.

Notably, in all of the experimental approaches, the 385*cyc8* ^−^ strain produced a significantly higher amount of carotenoids than did the wild-type and 385*tup1* ^−^ strains. Additionally, at the transcriptional level, the *cyc8* ^−^ mutation produced a stronger effect than did the *tup1* ^−^ mutation. These observations suggest that the two components of the co-repressor complex have different functions in the regulation of carotenogenesis. This is not surprising because it has been previously reported in *S. cerevisiae* and in other microorganisms that the repression activity of each component of the co-repressor complex may vary depending on the target gene. For example, unlike the *TUP1* gene, the *CYC8* gene is essential for viability in *Schizosaccharomyces pombe* [44–46]. In addition based on reports that Cyc8 forms a bridge between the DNA-binding regulator and Tup1 [44, 47], Tup1 may not be recruited to the target promoters in the absence of Cyc8. In that case, the repressive function of Tup1 would also be lost in the mutant strain 385*cyc8* ^−^. Thus, it would be expected that the *cyc8* ^−^ mutation would have a greater effect than *tup1* ^−^ on carotenogenesis if both gene products are involved in regulating this process. Under the same scenario, Cyc8 remains intact in the mutant 385*tup1* ^−^; thus, it can exert a residual repression by interacting with DNA-binding regulators such as Mig1. Thus, it is expected that the *tup1* ^−^ mutation would have a milder effect on carotenogenesis than the *cyc8* ^−^ mutation. In support of this idea, it has been demonstrated that the *mig1* ^−^ mutation caused a loss of glucose-mediated repression of carotenogenic genes and favored carotenoid production [29]. Considering that the regulator Mig1 requires the co-repressor Cyc8–Tup1 complex to exert the repressor function, the greater effect observed in the *mig1* ^−^ mutant strain than in 385*cyc8* ^−^ or 385*tup1* ^−^ regarding transcriptional repression of carotenogenic genes is expected as the *mig1* ^−^ mutation would simultaneously prevent the recruitment of both co-repressors at the target genes repressed by catabolic repression in *X. dendrorhous*.

In addition, it has been reported that once the repressor signal has ceased, Tup1 or Cyc8 may function as activators by recruiting other co-activators [48, 49]; thus, it is possible that the lower carotenoid production in the 385*tup1* ^−^ mutant strain than in the 385*cyc8* ^−^ strain may be due to a reduced recruitment of undescribed co-activators necessary for carotenoid production. On the other hand, although increases in the production of carotenoids were observed in the 385*cyc8* ^−^ and 385*tup1* ^−^ mutant strains, they did not reach the production levels observed in other deregulated carotenoid-overproducing strains obtained by random mutagenesis, which produced carotenoids even in the presence of glucose [50]. This suggests that in addition to catabolic repression, there may be other mechanisms that regulate carotenogenesis at different levels. This would not be surprising because in other yeasts, glucose affects the stability of some mRNAs, the translation rate, and the activity of some enzymes [21, 25, 42, 51].

### Identification of potential glucose-regulated genes in *X. dendrorhous* by bioinformatic transcriptomic analyses of the wild-type, 385*cyc8*^−^ and 385*tup1*^−^ strains

To identify possible genes regulated by glucose, the transcriptomes from the wild-type, 385*cyc8* ^−^ and 385*tup1* ^−^ strains obtained from RNA samples from cultures in MMv supplemented with glucose were analyzed and compared.

First, differentially expressed genes (DEGs) between the transcriptomes of the different strains were identified using the DESeq 2, EBSeq and EdgeR statistical methods, which were validated in a previous study [52]. Using the DESeq 2, EBSeq and EdgeR methods, respectively, 137, 422 and 1584 DEGs were identified in strain 385*cyc8* ^−^ and 121, 1008 and 1583 DEGs in strain 385*tup1* ^−^. Almost all of the DEGs that were identified with the strictest method, DESeq 2, coincided with those identified using the other two methods. Considering only the results obtained using all three of these statistical methods, a total of 136 and 119 DEGs were found in transcriptomes from strains 385*cyc8* ^−^ and 385*tup1* ^−^ when compared to the wild-type strain. Among the 136 DEGs identified in the 385*cyc8* ^−^ transcriptome, 128 were overexpressed and 8 were underexpressed compared to the wild-type. In the case of strain 385*tup1* ^−^, 44 and 75 DEGs were over- and underexpressed, respectively, compared to the wild-type. Using the results from both mutant strains, a total of 172 overexpressed DEGs were identified. In *S. cerevisiae*, approximately 180 genes are under the regulation of the Cyc8–Tup1 complex through its interaction with various DNA-binding transcription factors [24]. Thus, the number of genes whose expression is upregulated in the *X. dendrorhous cyc8* ^−^ and *tup1* ^−^ mutants is consistent with observations in other yeasts, even though the effects observed for some genes may be an indirect consequence of these mutations. Since this study focused on the repressor function of Cyc8 and Tup1, only the identified overexpressed DEG sequences in both mutant strains were analyzed using the BLASTx tool from NCBI to identify the potential gene products and their functions. Interestingly, the two mutants shared only one overexpressed DEG whose function could not be predicted by BLAST analyses. Among the 128 overexpressed DEGs in strain 385*cyc8* ^−^, only 49 had a greater than 30% identity with protein sequences available in the NCBI database (Additional file 5: Table S3). In the case of strain 385*tup1* ^−^, only 19 of the 44 overexpressed DEGs showed a high identity with proteins of known function (Additional file 6: Table S4). In both cases, the major group of gene products are involved in transmembrane transport, particularly those identified as carriers belonging to the MFS (Major Facilitator Superfamily) family, highlighting sugar and amino acid transporters and some transporters associated with drug resistance. In the case of strain 385*cyc8* ^−^, genes involved in transcriptional regulation, such as nit-4 or PacC, which are involved in nitrate assimilation [53] and pH response [54], respectively, were overexpressed in relation to the wild-type. Furthermore, potential genes involved in carbohydrate metabolism and stress response were also identified in this strain, which was expected because genes involved in the utilization of sugars or polysaccharides other than glucose and in the responses to various types of stresses are known targets of Cyc8 and Tup1 [21]. Similarly, overexpressed DEGs that would encode enzymes involved in carbohydrate metabolism and stress response, such as enzymes involved in trehalose metabolism, a universal stress protein and enzymes involved in DNA repair, were identified in strain 385*tup1* ^−^. Unlike strain 385*cyc8* ^−^, in strain 385*tup1* ^−^, the methylsterol monooxigenase-encoding gene (*ERG25*), which would be involved in ergosterol biosynthesis, was identified as an overexpressed DEG by all three statistical methods. This is an interesting result because ergosterol biosynthesis competes with carotenogenesis for the same precursors; thus, the lower production of carotenoids in strain 385*tup1* ^−^ compared to that in strain 385*cyc8* ^−^ may be because the synthesis of ergosterol is also favored by *ERG25* overexpression.

Focusing on genes that would be involved in the mevalonate pathway, which synthetizes the carotenogenesis precursors [55], the EBSeq 2 method identified the mevalonate kinase-encoding gene as an overexpressed DEG in both mutant strains and identified the hydroxy methyl glutaryl synthase-encoding gene (HMGS) as such only in strain 385*tup1* ^−^. In contrast, the EdgeR method identified the mevalonate kinase-encoding gene as an overexpressed DEG only in strain 385*cyc8* ^−^. In the case of the carotenogenic genes, the *crtS* gene was identified as an overexpressed DEG in both strains by the EBSeq method. Similarly, when using the RPKM value as a criterion, the *crtS* gene was approximately twofold overrepresented in both mutant strains (*crtS* RPKM values: 119 and 116 in strains 385*cyc8* ^−^ and 385*tup1* ^−^, respectively) compared to the wild-type (*crtS* RPKM value: 58), suggesting that, in addition to the carotenogenic pathway per se, Cyc8 and Tup1 also regulate the synthesis of carotenogenic precursors.

Many of the identified genes that were overexpressed in the *X. dendrorhous cyc8* ^−^ or *tup1* ^−^ mutants may be potential Mig1 targets because many of them are related to carbohydrate metabolism and sugar transport [21, 56]. The other possible regulators that recruit the Cyc8–Tup1 complex to the gene targets include Rox1 and Crt1, which regulate the expression of genes involved in the hypoxic response and of genes induced by DNA damage, respectively [26, 57, 58]. Consistent with this, in both mutant strains, some of the identified overexpressed genes are involved in DNA damage repair and may be regulated by Crt1. On the other hand, previous studies have shown that in *S. cerevisiae,* the combination of the regulator Rox1 and the Cyc8–Tup1 complex represses the expression of genes involved in the synthesis of ergosterol [59] and of other genes that encode enzymes that use oxygen as a substrate, including enzymes involved in sterol, fatty acid and heme group biosynthesis [58]. Consistent with this scenario, in addition to *ERG25* (mentioned above), which is involved in sterol biosynthesis, the overexpressed DEGs in strain 385*tup1* ^−^ included the coproporphyrinogen III oxidase (*HEM13*) gene, which is involved in the synthesis of heme.

In summary, the results obtained in this work indicate that *X. dendrorhous* has a functional catabolic repression mechanism in which the identified *CYC8* and *TUP1* genes are involved. The functionality of both genes in this mechanism was demonstrated by heterologous complementation in *S. cerevisiae* and by constructing *X. dendrorhous* mutants. In the *X. dendrorhous* mutants, several aspects of catabolic repression were affected, and the expression of genes known to be regulated by this mechanism in other organisms was altered. The results from this work strongly suggest that the identified *CYC8* and *TUP1* genes encode the components of the Cyc8–Tup1 co-repressor complex involved in catabolic repression and in other regulatory mechanisms, regulating the transcription of various groups of genes, including some carotenogenic genes, in *X. dendrorhous*.

## Conclusions

The *CYC8* and *TUP1* genes from *X. dendrorhous* identified in this work are functional, and their gene products are involved in the transcriptional regulation of several groups of genes regulated by catabolic repression, including some genes involved in the synthesis of carotenoids and carotenogenesis precursors in this yeast.

## Methods

### Strains and culture conditions

All of the strains that were used and obtained in this work are described in Table 1. *Xanthophyllomyces dendrorhous* mutant strains derive from the wild-type strain UCD 67–385 (ATCC 24230) and were obtained via homologous recombination to replace the corresponding gene with a cassette that confers resistance to an antibiotic [39]. *Xanthophyllomyces dendrorhous* strains were cultured at 22 °C in YM medium (0.3% yeast extract, 0.3% malt extract and 0.5% peptone) supplemented in some cases with glucose (10 g l^−1^) or in Vogel minimum medium (MMv) supplemented with 1 or 2% glucose, 2% maltose or 1% glycerol [17]. Antibiotic-resistant yeast strains were cultured in YM medium supplemented with hygromycin B and/or zeocin at final concentrations of 15 and 20 µg ml^−1^, respectively. The *S. cerevisiae* strains were cultured at 22 or 30 °C in SD minimum medium (0.67% Yeast Nitrogen Base (Difco™, Becton, Dickinson and Company, Franklin Lakes, NJ, USA) and 2% glucose or 2% sucrose as indicated), supplemented with uracil (0.02 g l^−1^), histidine (0.02 g l^−1^), methionine (0.02 g l^−1^) or leucine (0.1 g l^−1^) when necessary based on the strain auxotrophy.

Cell growth was determined by measuring the optical density of the cultures at 600 nm (OD_600_). *X. dendrorhous* and *S. cerevisiae* growth curves were initiated at an OD_600_ of 0.1 and were performed in triplicate. Growth parameters, such as maximum growth rate (μ) and generation time (gt), were estimated based on the Gompertz model of microbial growth [60] using a minimum of five data points in each case. Biomass was quantified by measuring the dry weight of yeast in 1 ml of culture in triplicate using an analytical balance. The DNS method [61] was used to quantify the reducing sugars, such us glucose or maltose, remaining in the medium. For this, the medium from a culture sample was mixed 1:1 with the DNS reagent (1% DNS, 1.6% NaOH and 15% Na–K tartrate) and heated at 100 °C for 10 min. Subsequently, the reaction was stopped on ice, and the absorbance at 540 nm was measured. To determine the sugar concentration, a standard curve (absorbance at 540 nm vs. sugar concentration) was constructed using aqueous solutions with known sugar concentrations between 0.05 and 0.5 g l^−1^.

### Plasmids, DNA extraction and amplification

All of the plasmids that were used and constructed in this work are listed in Table 1. Oligonucleotides were synthesized by *Integrated DNA Technologies* (IDT, Coralville, IA, USA) and are described in Table 2.

The plasmid pgTUP1 was constructed by inserting at the *Eco*RV site of pBluescript II SK (−) XR a 2.7 kb DNA fragment of the *TUP1* gene that was previously amplified using the primers Tup1g1Rv and Tup1C2Fw and using genomic DNA from strain UCD 67–385 as a template. Subsequently, pgTUP1 was digested with *Eco*RV to remove 2003 bp of the *TUP1* gene. Finally, a module that confers resistance to the antibiotics hygromycin B or zeocin was ligated to the digested vector, generating plasmids pgTUP1-Hyg and pgTUP1-Zeo, respectively. To transform *X. dendrorhous*, transformant DNA was released from the plasmids pgTUP1-Hyg and pgTUP1-Zeo by digestion with *Ava*I and *Xho*I, respectively (Additional file 7: Figure S3).

The plasmid pgCYC8 was obtained by inserting at the *Eco*RV site of pBluescript II SK (−) XR a 2.4 kb PCR product of the *CYC8* gene that was previously amplified using the primers GTR1Fwd and GTR1Rev and using genomic DNA from strain UCD 67–385 as a template. Then, the module that confers resistance to hygromycin B was inserted at the *Bmg*BI site to yield the plasmid pgCYC8-Hyg, and the zeocin resistance module was inserted between the *Bmg*BI and *Pml*I sites of plasmid pgCYC8, resulting in plasmid pgCYC8-Zeo. For *X. dendrorhous* transformation, plasmids pgCYC8-Hyg and pgCYC8-Zeo were digested with *Ava*I and *Bam*HI, respectively, to release the transformant DNA (Additional file 7: Figure S3).

To construct the plasmids YEp-NP-CYC8 and YEp-NP-TUP1, the *CYC8* and *TUP1* ORFs were amplified from strain UCD 67–385 cDNA using the primer pairs Tup1C1fw + Tup1C1Rv and GTRORF_ATGfw + GTRORF_TGARv, respectively. The amplification products were inserted at the *Xba*I site of the expression vector YEp-NP, and the desired orientation was confirmed by PCR.

Plasmid DNA extraction from *Escherichia coli* and purification was performed using the AxyPrep™ Plasmid Miniprep and Midiprep Kits (Axygen^®^, Corning Incorporated, Corning, NY, USA) according to the manufacturer’s instructions. Total DNA extraction from *X. dendrorhous* and *S. cerevisiae* was performed as previously described [62].

Standard PCR reactions, restriction enzyme digestions, ligation reactions and *E. coli* transformations were performed according to standard protocols described in the Molecular Cloning Manual [37]. The *Pfu* DNA polymerase (Stratagene, Agilent Technologies Inc, Santa Clara, CA, USA), RNase A (US Biological, Salem, MA, USA), T4 DNA ligase (Fermentas, Thermo Fisher Scientific Inc, Waltham, MA, USA) and restriction endonucleases were used according to the manufacturers’ instructions.

The integrity and concentration of chromosomal DNA, plasmids, RNA and PCR products were analyzed by using 0.7–1% agarose gel electrophoresis in 1X TAE buffer (40 mM Tris–acetate, 1 mM EDTA, pH 8.0) stained with ethidium bromide 0.5 µg ml^−1^. Purification of DNA fragments from agarose gels was performed using the Ultra Clean™ 15 DNA Purification Kit (MO BIO Laboratories Inc., Carlsbad, CA, USA) following the manufacturer’s recommended protocol.

### Transformation by electroporation

*Xanthophyllomyces dendrorhous* electrocompetent cells were obtained from cultures in the exponential phase of growth (OD_600_: 2–4) developed in YM medium as previously described [63]. Cells were electroporated using a Bio-Rad Gene Pulser X-Cell with PC and CE modules (BIORAD, Hercules, CA, USA) under the following conditions: 125 mF, 600 Ω, and 0.45 kV. Transformants were confirmed as *X. dendrorhous* through an analysis of the Intergenic Spacer (IGS) region between the LrDNA and 5S genes [64], which was amplified and sequenced using primers LR12R and 5SRNA.

*Saccharomyces cerevisiae* electrocompetent cells were obtained from exponentially growing cultures in YEP medium at 22 °C with constant agitation. Cells were washed with chilled water and then with 1 M sorbitol. Then, cells were suspended in 0.2 ml of 1 M sorbitol, and 40 μl aliquots were used for electroporation under the following conditions: 1.5 kV, 25 μF and 200 Ω.

### Extracellular invertase activity

The extracellular invertase activity was measured as described previously [65], allowing the quantification of the glucose that is released from sucrose (an invertase substrate). In the case of *X. dendrorhous*, aliquots of culture supernatant were taken and analyzed by mixing 1:1 with 50 mM sodium acetate buffer pH 5.4 with 2% sucrose. As a control, a fraction of each sample was mixed with the same buffer without including sucrose. Mixtures were incubated at 45 °C for 30 min, and then the reaction was stopped by incubation at −20 °C. For the *S. cerevisiae* assays, direct aliquots of cultures were taken and mixed 1:1 with 50 mM sodium acetate buffer pH 5.4 with or without 2% sucrose and then incubated at 30 °C for 30 min. In both cases, the glucose released during the reaction was determined using the DNS method [61]. In each case, three culture samples were taken, and three independent measurements were performed, from which an average was obtained. Finally, the activity was normalized to the dry weight of the sample and is expressed in (g of glucose) (g of yeast dry weight)^−1^.

### Carotenoid extraction and RP-HPLC analysis

*Xanthophyllomyces dendrorhous* total carotenoids were obtained by acetone extraction [66]. Carotenoids were quantified spectrophotometrically at 474 nm using an absorption coefficient of A1% = 2100 and normalized to the yeast dry weight. The carotenoid composition was analyzed by reverse-phase liquid chromatography (RP-HPLC) using a LiChrospher RP18 125-4 (Merck Millipore, Billerica, MA USA) column and acetonitrile:methanol:isopropanol (85:10:5) as the mobile phase with a 1 ml min^−1^ flow rate under isocratic conditions. Each pigment was identified by comparison with specific standards (Sigma, San Luis, Missouri, USA) based on retention time and absorption spectra [67] using a Shimadzu SPD-M10A diode array detector (Shimadzu, Kyoto, Japan).

### RNA extraction, cDNA synthesis and qPCR

*Xanthophyllomyces dendrorhous* total RNA was extracted using the Yeast RiboPure kit (Life Technologies, Thermo Fisher Scientific Inc, Waltham, MA, USA) according to the manufacturer’s recommendations. cDNA synthesis was performed with 5 µg of total RNA, oligo-dT18, 1.25 µM dNTPs and 200 U of reverse transcriptase M-MLV (Invitrogen™, Thermo Fisher Scientific Inc., Waltham, MA, USA) in a final volume of 20 µl, according to the protocol recommended by the supplier. Determination of the relative transcript levels was performed using a M × 3000P quantitative PCR system (Stratagene, Agilent Technologies Inc., Santa Clara, CA, USA) and analyzed by mixing 1 μl of the reverse transcription reaction, 0.25 μM of each primer and 10 μl of the SensiMix™ SYBR^®^ Green I kit (Bioline, London, UK) in a final volume of 20 μl. The obtained Ct values were normalized to the values for β-actin, encoded by *ACT* [GenBank: X89898.1] [68], and subsequently expressed as a function of the control conditions using the 2^−∆∆Ct^ algorithm [69].

### Sequencing and bioinformatic analyses

DNA sequencing was performed using an automatic sequencer (ABI PRISM 3100 Genetic Analyzer, Applied Biosystems, Thermo Fisher Scientific Inc, Waltham, MA, USA) with fluorescent terminators (BigDye Terminator Kit v3.1, Applied Biosystems, Thermo Fisher Scientific Inc., Waltham, MA, USA). A minimum of 200 or 50 ng of total DNA was used for sequencing plasmids or purified DNA fragments, respectively.

Sequence analyses used Geneious 8.1.3 and online tools, such as BLAST or ORF Finder of the National Center for Biotechnology Information (NCBI; http://www.ncbi.nlm.nih.gov). Multiple sequence alignments were performed using the ClustalW2 online tool (www.ebi.ac.uk/Tools/msa/clustalw2) and edited using the program Jalview, downloaded from www.jalview.org.

In addition, genomic and transcriptomic sequences of the UCD 67-385 wild-type strain available in our laboratory were analyzed using CLC Genomics Workbench 5 as previously described [30].

Transcriptomes of the UCD 67–385 (wild-type), 385*cyc8* ^−^ and 385*tup1* ^−^ strains were obtained. For this purpose, the three strains were cultured in MMv supplemented with 2% glucose as the sole carbon source. After 36 h of culture at 22 °C, a time point at which the residual glucose in the culture medium was still high (10 g l^−1^, approximately), cells were harvested, and total RNA was then extracted from yeast pellets. The RNA samples were sequenced at Macrogen, Inc. (Seoul, South Korea) using the Illumina HiSeq 2000 System, including a 100 bp paired-end library as described previously [30]. The assembly and analysis of the three obtained transcriptomes were performed using the CLC Genomics Workbench 5 program.

Additionally, the programs R (downloaded from https://cran.r-project.org) and R studio (downloaded from www.rstudio.com) were used for statistical analyses of the RNA-seq data. The DESeq 2, EBSeq and EgdeR packages obtained from Bioconductor software (https://bioconductor.org) were used according to the user manuals to identify differentially expressed genes (DEGs) among the different transcriptomes using the default parameters in all cases. Additionally, the transcript levels of some genes were estimated from the numbers of reads in each transcriptome and are expressed using the RPKM (Reads Per Kilobase per Million Mapped reads) value, which mirrors the molar concentration of each transcript in the starting sample normalized by the length of the RNA and the total number of reads [70].

## Additional files

**Additional file 1: Table S1.** Primers used in this work. Hybridization targets, sequences and orientations of the primers used in the study.

**Additional file 2: Table S2.** Carotenoid composition in *X. dendrorhous* wild-type and mutant strains cultured in MMv with 2% glucose. Analysis of carotenoid composition at different growth phases in *X. dendrorhous* wild-type, 385*cyc8* ^−^ and 385*tup1* ^−^ strains.

**Additional file 3: Figure S1.** HPLC analysis of carotenoid composition of *X. dendrorhous* wild-type, 385*cyc8* ^−^ and 385*tup1* ^−^ strains. For each strain, a representative sample obtained at the late stationary phase of growth is shown. Values are the percentage representativeness of each compound with respect to the total carotenoid content.

**Additional file 4: Figure S2.** Glucose effect on transcript levels of genes involved in carotenogenesis. The expression kinetics at the mRNA level after the addition of glucose (final concentration of 20 g l^−1^) was determined relative to control (without glucose) for different genes involved in carotenogenesis (early stages: *HMGR*, *idi*, *FPS* and *crtE*; final stages: *crtI*, *crtYB*, *crtS* and *crtR*) and genes used as controls (*INV* and *grg2*) in the *X. dendrorhous* wild-type, 385*cyc8* ^−^ and 385*tup1* ^−^ strains. The horizontal axis of the graphs was set at 1. The data represent the average of three independent experiments, and the bars represent the standard error.

**Additional file 5: Table S3.** Identified overexpressed DEGs (according to the DESeq 2 results) in strain 385*cyc8* ^−^. Results of the BLAST analysis of the ORF sequences identified as overexpressed DEGs in strain 385*cyc8* ^−^.

**Additional file 6: Table S4.** Identified overexpressed DEGs (according to the DESeq 2 results) in strain 385*tup1* ^−^. Results of the BLAST analysis of the ORF sequences identified as overexpressed DEGs in strain 385*tup1* ^−^.

**Additional file 7: Figure S3.** Plasmids used for *X. dendrorhous* transformation. Up: pgCYC8-Hyg and pgCYC8-Zeo plasmids used for 385*cyc8* ^−^ mutant strain construction. Down: pgTUP1-Hyg and pgTUP1-Zeo plasmids used for 385*tup1* ^−^ mutant strain construction. The restriction sites used to release the transformant DNA are shown. pEF: elongation factor 1α promoter (*X. dendrorhous*); *GPDH*t: glyceraldehyde 3 phosphate dehydrogenase terminator (*X. dendrorhous*). *hph*: hygromycin phosphotransferase gene (*E. coli*); *ble*: *ble* gene (S*treptoalloteichus hindustanus)*; AmpR: ampicillin resistance; pUC ori: pUC replication origin.

## Abbreviations

PBS: phytoene-β-carotene synthase
IPP: isopentenyl pyrophosphate
DMAPP: dimethylallyl pyrophosphate
GGPP: geranylgeranyl pyrophosphate
FPP: farnesyl pyrophosphate
DEGs: differentially expressed genes
RPKM: reads per kilobase per million mapped reads
ORF: open reading frame
MFS: major facilitator superfamily
RP-HPLC: reverse-phase liquid chromatography
IGS: intergenic spacer
DNS: dinitrosalicylic acid
MMv: Vogel minimum medium
2DG: 2-deoxy-d-glucose

## References

[1] Rodríguez-Sáiz M, de la Fuente JL, Barredo JL (2010). *Xanthophyllomyces dendrorhous* for the industrial production of astaxanthin. Appl Microbiol Biotechnol.

[2] Schmidt I, Schewe H, Gassel S, Jin C, Buckingham J, Hümbelin M, Sandmann G, Schrader J (2011). Biotechnological production of astaxanthin with *Phaffia rhodozyma*/*Xanthophyllomyces dendrorhous*. Appl Microbiol Biotechnol.

[3] Miller MW, Yoneyama M, Soneda M (1976). *Phaffia*, a new yeast genus in the Deuteromycotina (Blastomycetes). Int J Syst Bacteriol.

[4] Libkind D, Moline M, de Garcia V, Fontenla S, van Broock M (2008). Characterization of a novel South American population of the astaxanthin producing yeast *Xanthophyllomyces dendrorhous* (*Phaffia rhodozyma*). J Ind Microbiol Biotechnol.

[5] Weber RW, Becerra J, Silva MJ, Davoli P (2008). An unusual *Xanthophyllomyces* strain from leaves of *Eucalyptus globulus* in Chile. Mycol Res.

[6] Contreras G, Barahona S, Sepulveda D, Baeza M, Cifuentes V, Alcaíno J (2015). Identification and analysis of metabolite production with biotechnological potential in *Xanthophyllomyces dendrorhous* isolates. World J Microbiol Biotechnol.

[7] Loto I, Gutierrez MS, Barahona S, Sepulveda D, Martinez-Moya P, Baeza M, Cifuentes V, Alcaíno J (2012). Enhancement of carotenoid production by disrupting the C22-sterol desaturase gene (*CYP61*) in *Xanthophyllomyces dendrorhous*. BMC Microbiol.

[8] Hara KY, Morita T, Mochizuki M, Yamamoto K, Ogino C, Araki M, Kondo A (2014). Development of a multi-gene expression system in *Xanthophyllomyces dendrorhous*. Microb Cell Fact.

[9] Kajiwara S, Fraser PD, Kondo K, Misawa N (1997). Expression of an exogenous isopentenyl diphosphate isomerase gene enhances isoprenoid biosynthesis in *Escherichia coli*. Biochem J.

[10] Alcaíno J, Romero I, Niklitschek M, Sepúlveda D, Rojas MC, Baeza M, Cifuentes V (2014). Functional characterization of the *Xanthophyllomyces dendrorhous* farnesyl pyrophosphate synthase and geranylgeranyl pyrophosphate synthase encoding genes that are involved in the synthesis of isoprenoid precursors. Plos ONE.

[11] Verdoes JC, Krubasik P, Sandmann G, Van Ooyen AJJ (1999). Isolation and functional characterisation of a novel type of carotenoid biosynthetic gene from *Xanthophyllomyces dendrorhous*. Mol Gen Genet.

[12] Verdoes JC, Misawa N, van Ooyen AJ (1999). Cloning and characterization of the astaxanthin biosynthetic gene encoding phytoene desaturase of *Xanthophyllomyces dendrorhous*. Biotechnol Bioeng.

[13] Alvarez V, Rodriguez-Saiz M, de la Fuente JL, Gudina EJ, Godio RP, Martin JF, Barredo JL (2006). The *crtS* gene of *Xanthophyllomyces dendrorhous* encodes a novel cytochrome-P450 hydroxylase involved in the conversion of beta-carotene into astaxanthin and other xanthophylls. Fungal Genet Biol.

[14] Ojima K, Breitenbach J, Visser H, Setoguchi Y, Tabata K, Hoshino T, van den Berg J, Sandmann G (2006). Cloning of the astaxanthin synthase gene from *Xanthophyllomyces dendrorhous* (*Phaffia rhodozyma*) and its assignment as a β-carotene 3-hydroxylase/4-ketolase. Mol Genet Genom.

[15] Alcaíno J, Barahona S, Carmona M, Lozano C, Marcoleta A, Niklitschek M, Sepulveda D, Baeza M, Cifuentes V (2008). Cloning of the cytochrome p450 reductase (*crtR*) gene and its involvement in the astaxanthin biosynthesis of *Xanthophyllomyces dendrorhous*. BMC Microbiol.

[16] Marcoleta A, Niklitschek M, Wozniak A, Lozano C, Alcaíno J, Baeza M, Cifuentes V (2011). Glucose and ethanol-dependent transcriptional regulation of the astaxanthin biosynthesis pathway in *Xanthophyllomyces dendrorhous*. BMC Microbiol.

[17] Wozniak A, Lozano C, Barahona S, Niklitschek M, Marcoleta A, Alcaíno J, Sepulveda D, Baeza M, Cifuentes V (2011). Differential carotenoid production and gene expression in *Xanthophyllomyces dendrorhous* grown in a nonfermentable carbon source. FEMS Yeast Res.

[18] Dowzer CE, Kelly JM (1991). Analysis of the *creA* gene, a regulator of carbon catabolite repression in *Aspergillus nidulans*. Mol Cell Biol.

[19] Linde D, Macias I, Fernandez-Arrojo L, Plou FJ, Jimenez A, Fernandez-Lobato M (2009). Molecular and biochemical characterization of a beta-fructofuranosidase from *Xanthophyllomyces dendrorhous*. Appl Environ Microbiol.

[20] Gutiérrez-Alonso P, Gimeno-Pérez M, Ramírez-Escudero M, Plou FJ, Sanz-Aparicio J, Fernández-Lobato M (2016). Molecular characterization and heterologous expression of a *Xanthophyllomyces dendrorhous* α-glucosidase with potential for prebiotics production. Appl Microbiol Biotechnol.

[21] Rolland F, Winderickx J, Thevelein JM (2002). Glucose-sensing and-signalling mechanisms in yeast. FEMS Yeast Res.

[22] Wong KH, Struhl K (2011). The Cyc8–Tup1 complex inhibits transcription primarily by masking the activation domain of the recruiting protein. Genes Dev.

[23] Roy A, Jouandot D, Cho KH, Kim JH (2014). Understanding the mechanism of glucose-induced relief of Rgt1-mediated repression in yeast. FEBS Open Bio.

[24] Smith RL, Johnson AD (2000). Turning genes off by Ssn6-Tup1: a conserved system of transcriptional repression in eukaryotes. Trends Biochem Sci.

[25] Gancedo JM (1998). Yeast carbon catabolite repression. Microbiol Mol Biol Rev.

[26] Zhang Z, Reese JC (2005). Molecular genetic analysis of the yeast repressor Rfx1/Crt1 reveals a novel two-step regulatory mechanism. Mol Cell Biol.

[27] Galdieri L, Mehrotra S, Yu S, Vancura A (2010). Transcriptional regulation in yeast during diauxic shift and stationary phase. OMICS.

[28] Liu YS, Wu JY (2008). Modeling of *Xanthophyllomyces dendrorhous* growth on glucose and overflow metabolism in batch and fed-batch cultures for astaxanthin production. Biotechnol Bioeng.

[29] Alcaíno J, Bravo N, Córdova P, Marcoleta AE, Contreras G, Barahona S, Sepúlveda D, Fernández-Lobato M, Baeza M, Cifuentes V (2016). The involvement of Mig1 from *Xanthophyllomyces dendrorhous* in catabolic repression: an active mechanism contributing to the regulation of carotenoid production. Plos ONE.

[30] Baeza M, Alcaíno J, Barahona S, Sepúlveda D, Cifuentes V (2015). Codon usage and codon context bias in *Xanthophyllomyces dendrorhous*. BMC Genom.

[31] Loubradou G, Brachmann A, Feldbrügge M, Kahmann R (2001). A homologue of the transcriptional repressor Ssn6p antagonizes cAMP signalling in *Ustilago maydis*. Mol Microbiol.

[32] Olmedo M, Navarro-Sampedro L, Ruger-Herreros C, Kim S-R, Jeong B-K, Lee B-U, Corrochano LM (2010). A role in the regulation of transcription by light for RCO-1 and RCM-1, the *Neurospora* homologs of the yeast Tup1-Ssn6 repressor. Fungal Genet Biol.

[33] D’Andrea LD, Regan L (2003). TPR proteins: the versatile helix. Trends Biochem Sci.

[34] Trumbly RJ (1988). Cloning and characterization of the *CYC8* gene mediating glucose repression in yeast. Gene.

[35] Jabet C, Sprague ER, VanDemark AP, Wolberger C (2000). Characterization of the N-terminal domain of the yeast transcriptional repressor Tup1. J Biol Chem.

[36] Zhang Z, Varanasi U, Trumbly RJ (2002). Functional dissection of the global repressor Tup1 in yeast: dominant role of the C-terminal repression domain. Genetics.

[37] Sambrook J, Russell DW (2001). Molecular cloning: a laboratory manual.

[38] Winzeler EA, Shoemaker DD, Astromoff A, Liang H, Anderson K, Andre B, Bangham R, Benito R, Boeke JD, Bussey H, Chu AM, Connelly C, Davis K, Dietrich F, Dow SW, El Bakkoury M, Foury F, Friend SH, Gentalen E, Giaever G, Hegemann JH, Jones T, Laub MLH, Liebundguth N, Lockhart DJ, Lucau-Danila A, Lussier MMN, Menard P, Mittmann M, Pai CRC, Revuelta JLRL, Roberts CJ, Ross-MacDonald P, Scherens B, Snyder M, Sookhai-Mahadeo S, Storms RK, Véronneau S, Voet M, Volckaert G, Ward TR, Wysocki R, Yen GS, Yu K, Zimmermann K, Philippsen P, Johnston M, Davis RW (1999). Functional characterization of the *S. cerevisiae* genome by gene deletion and parallel analysis. Science.

[39] Niklitschek M, Alcaíno J, Barahona S, Sepulveda D, Lozano C, Carmona M, Marcoleta A, Martinez C, Lodato P, Baeza M, Cifuentes V (2008). Genomic organization of the structural genes controlling the astaxanthin biosynthesis pathway of *Xanthophyllomyces dendrorhous*. Biol Res.

[40] Sánchez-Torres P, González-Candelas L, Ramón D (1998). Heterologous expression of a *Candida molischiana* anthocyanin-beta-glucosidase in a wine yeast strain. J Agric Food Chem..

[41] Leiva K, Werner N, Sepulveda D, Barahona S, Baeza M, Cifuentes V, Alcaíno J (2015). Identification and functional characterization of the *CYP51* gene from the yeast *Xanthophyllomyces dendrorhous* that is involved in ergosterol biosynthesis. BMC Microbiol.

[42] Trumbly RJ (1992). Glucose repression in the yeast *Saccharomyces cerevisiae*. Mol Microbiol.

[43] Williams FE, Trumbly RJ (1990). Characterization of *TUP1*, a mediator of glucose repression in *Saccharomyces cerevisiae*. Mol Cell Biol.

[44] Tzamarias D, Struhl K (1995). Distinct TPR motifs of Cyc8 are involved in recruiting the Cyc8–Tup1 corepressor complex to differentially regulated promoters. Genes Dev.

[45] Cooper JP, Roth SY, Simpson RT (1994). The global transcriptional regulators, SSN6 and TUP1, play distinct roles in the establishment of a repressive chromatin structure. Genes Dev.

[46] Fagerström-Billai F, Durand-Dubief M, Ekwall K, Wright AP (2007). Individual subunits of the Ssn6-Tup11/12 corepressor are selectively required for repression of different target genes. Mol Cell Biol.

[47] Palaiomylitou M, Tartas A, Vlachakis D, Tzamarias D, Vlassi M (2008). Investigating the structural stability of the Tup1-interaction domain of Ssn6: evidence for a conformational change on the complex. Proteins: Struct, Funct, Bioinf.

[48] Conlan RS, Gounalaki N, Hatzis P, Tzamarias D (1999). The Tup1–Cyc8 protein complex can shift from a transcriptional co-repressor to a transcriptional co-activator. J Biol Chem.

[49] Proft M, Struhl K (2002). Hog1 kinase converts the Sko1–Cyc8–Tup1 repressor complex into an activator that recruits SAGA and SWI/SNF in response to osmotic stress. Mol Cell.

[50] Lodato P, Alcaíno J, Barahona S, Retamales P, Jimenez A, Cifuentes V (2004). Study of the expression of carotenoid biosynthesis genes in wild-type and deregulated strains of *Xanthophyllomyces dendrorhous* (Ex.: *Phaffia rhodozyma*). Biol Res.

[51] Horak J, Wolf DH (1997). Catabolite inactivation of the galactose transporter in the yeast *Saccharomyces cerevisiae*: ubiquitination, endocytosis, and degradation in the vacuole. J Bacteriol.

[52] Soneson C, Delorenzi M (2013). A comparison of methods for differential expression analysis of RNA-seq data. BMC Bioinform.

[53] Yuan GF, Fu YH, Marzluf G (1991). nit-4, a pathway-specific regulatory gene of *Neurospora crassa*, encodes a protein with a putative binuclear zinc DNA-binding domain. Mol Cell Biol.

[54] Caracuel Z, Roncero MIG, Espeso EA, González-Verdejo CI, García-Maceira FI, Di Pietro A (2003). The pH signalling transcription factor PacC controls virulence in the plant pathogen *Fusarium oxysporum*. Mol Microbiol.

[55] Sharma R, Gassel S, Steiger S, Xia X, Bauer R, Sandmann G, Thines M (2015). The genome of the basal agaricomycete *Xanthophyllomyces dendrorhous* provides insights into the organization of its acetyl-CoA derived pathways and the evolution of Agaricomycotina. BMC Genom.

[56] Zimmermann FK, Entian KD (1997). Yeast sugar metabolism biochemistry, genetics, biotechnology and applications.

[57] Huang M, Zhou Z, Elledge SJ (1998). The DNA replication and damage checkpoint pathways induce transcription by inhibition of the Crt1 repressor. Cell.

[58] Balasubramanian B, Lowry CV, Zitomer RS (1993). The Rox1 repressor of the *Saccharomyces cerevisiae* hypoxic genes is a specific DNA-binding protein with a high-mobility-group motif. Mol Cell Biol.

[59] Martínez F, Pascual-Ahuir A, Proft M (2011). Repression of ergosterol biosynthesis is essential for stress resistance and is mediated by the Hog1 MAP kinase and the Mot3 and Rox1 transcription factors. Mol Microbiol.

[60] Zwietering MH, Jongenburger I, Rombouts FM, Van’t Riet K (1990). Modeling of the bacterial growth curve. Appl Environ Microbiol.

[61] Miller GL (1959). Use of dinitrosalicylic acid reagent for determination of reducing sugar. Anal Chem.

[62] Cifuentes V, Hermosilla G, Martinez C, Leon R, Pincheira G, Jimenez A (1997). Genetics and electrophoretic karyotyping of wild-type and astaxanthin mutant strains of *Phaffia rhodozyma*. Antonie Van Leeuwenhoek.

[63] Adrio JL, Veiga M (1995). Transformation of the astaxanthin-producing yeast *Phaffia rhodozyma*. Biotechnol Tech.

[64] Fell JW, Blatt GM (1999). Separation of strains of the yeasts *Xanthophyllomyces dendrorhous* and *Phaffia rhodozyma* based on rDNA IGS and ITS sequence analysis. J Ind Microbiol Biotechnol.

[65] Goldstein A, Lampen JO (1975). ß-d-fructofuranoside fructohydrolase from yeast. Methods Enzymol.

[66] An G-H, Schuman DB, Johnson EA (1989). Isolation of *Phaffia rhodozyma* mutants with increased astaxanthin content. Appl Environ Microbiol.

[67] Mercadante AZ, Egeland ES (2004). Carotenoids Handbook.

[68] Wery J, Dalderup MM, Linde JT, Boekhout T, Van Ooyen AJ (1996). Structural and phylogenetic analysis of the actin gene from the yeast *Phaffia rhodozyma*. Yeast.

[69] Livak KJ, Schmittgen TD (2001). Analysis of relative gene expression data using real-time quantitative PCR and the 2^−∆∆*C*T^ method. Methods.

[70] Mortazavi A, Williams BA, McCue K, Schaeffer L, Wold B (2008). Mapping and quantifying mammalian transcriptomes by RNA-Seq. Nat Methods.

