# Supplementary material for: Regulation of carotenogenesis in the red yeast Xanthophyllomyces dendrorhous: the role of the transcriptional co-repressor complex Cyc8–Tup1 involved in catabolic repression
Source: Microb Cell Fact. 2016 Nov 14;15:193. doi: 10.1186/s12934-016-0597-1 (PMC5109733; doi:10.1186/s12934-016-0597-1)
Supplement: Supplementary file 1 — Additional file 1: Table S1. Primers used in this work. Hybridization targets, sequences and orientations of the primers used in the study. [file 12934_2016_597_MOESM1_ESM.pdf]

Table S1. Primers used in this work

Primer name	Hybridization target	Sequence (5' to 3')	Orientation
**GTRORF_ATGfw**	*CYC8* gene (*X. dendrorhous*)	ATGGCGATTGGAAACAGTGTAG	F
**GTRORF_TGARv**		TCAGGCTTTGACATCTTCCATTTC	R
**GTR_RT1Fwd**		GTGCCACCTCCGCCTCAAG	F
**GTR_RT2Fwd**		GGTACGGGATTGGTATTCTG	F
**GTR_RT3Fwd**		CACGGTGGAAGAAGATCTCC	F
**GTR_RT4Fwd**		GACATTCAGACACCTACCCTG	F
**GTR_RT5Fwd**		CGACTTCCGGTGGAAACAG	F
**GTR_RT6Fwd**		CTTCCATCTCTTTCTTCGCC	F
**GTR_RT7Fwd**		CTTCCCTTATTACTTCTTGCC	F
**GTR_RT1Rev**		CGCGGTCGGGTGAACGTC	R
**GTR_RT2Rev**		GAAAGCCTCCTCTGCATGC	R
**GTR_RT3Rev**		GGAACGGAGATCTGGCGCC	R
**GTR_RT5Rev**		CGACAGCGTCCGAATTAGAC	R
**GTR_RT6Rev**		GAGGCCGAAGCATTAGCAG	R
**GTR_RT7Rev**		GACACAACATATACACAGCCG	R
**Tup1C1fw**	*TUP1* gene (*X. dendrorhous*)	ATGCTCGACGCCGTCAAAAG	F
**Tup1C1Rv**		CTAATCCCTAACGGAATCATAC	R
**Tup1C2fw**		GATGCAGATCCAAAATAGCTCC	F
**Tup1 C3fw**		GGCACAAGAACTCCCACTCC	F
**Tup1 C4fw**		CGAACGATTTGTTTCTCCCC	F
**Tup1 C5fw**		GATACGTAGCAGCGGGTTCG	F
**Tup1 C6fw**		CCGTTGGAGTTTCAAATGAC	F
**Tup1C2Rv**		GCACACTTCTGTCCTTTGAAC	R
**Tup1C3Rv**		CGAGCTGAACACCCGTCTG	R
**Tup1C4Rv**		GTTTGAGAACTTGACGCAGC	R
**Tup1C5Rv**		AGTGAGTGGAGGAAGGCTG	R
**Tup1C6Rv**		GTCGCAGGTGAGAGATTTCG	R
**Tup1g1Rv**		CCATAGTTGGAAGATGATTAC	R
**Tup1g2Rv**		CCCAATCGCACTCACTTGAC	R
**Tup1g3fw**		CACGCATCCAGGCTCGGCG	F
**Tup1g3Rv**		CCCCGACCCTTGACCTCG	R
**M13Fw**	pBluescript II SK(-)XR	GTAAAACGACGGCCAG	F
**M13Rev**		GGAAACAGCTATGACCATG	R
**HF**	*hph* gene (*E. coli*)	ATGAAAAAGCCTGAACTCACC	F
**HR**		CTATTCCTTTGCCCTCGGAC	R
**ZF**	*ble* gene (*S. hindustanus*)	ATGGCCAAGTTGACCAGTGC	F
**ZR**		TCAGTCCTGCTCCTCGGCC	R
**zeoc-sense-Fw**		ACGACGTGACCCTGTTCATCA	F
**zeoc-antisense-Rv**		TGATGAACAGGGTCACGTCGT	R
**PEFForEV**	EF-1α promoter (*X. dendrorhous*)	GATATCGGCTCATCAGCCGACAGTT	F
**pef rev o**		TTTGAAGCTGTTCGAGATAG	R
**gpdTF**	*GPDH* terminator (*X. dendrorhous*)	ACGGTTCTCTCCAAACCCTC	F
**GPDHtRev**		ATCATGAGAGATGACGGAG	R
**TDH3t.F**	*TDH3* terminator (*S. cerevisiae*)	TCCCTCTGTGAGTGAATTTACTTTAAATC	F
**TDH3t.R**		ATCCTGGCGGAAAAAATTCATTTG	R
**OligodT^{18}**	PolyA region	TTTTTTTTTTTTTTTTTT	R
**OligodT^{30}**		TTTTTTTTTTTTTTTTTTTTTTTTTTTTTT	R
**LR12R**	rDNA 28S	CTGAACGCCTCTAAGTCAGAA	F
**5SRNA**	rDNA 5S	ATCAGACGGGATGCGGT	R
**Prmers used in RT-qPCR analyses.**			
**mactF-RT**	*ACT* gene (*X. dendrorhous*)	CCGCCCTCGTGATTGATAAC	F
**mactR-RT**		TCACCAACGTAGGAGTCCTT	R
**midif-RT**	*idi* gene (*X. dendrorhous*)	TCCGAACCGAAGGACTCAGTTT	F
**midir-RT**		GGACATCAAGTGGCAGGTCT	R
**mfpsf2-RT**	*FPS* gene (*X. dendrorhous*)	TGGTACAAAGTTGAGGGAGTGCT	F
**mfpsr2-RT**		AGCGGTCAACAGATCGATGAG	R

**mcrtEF-RT**	*crtE* gene (*X. dendrorhous*)	TGTTGGCATGCTACATACCG	F
**mcrtER-RT**		GTTGGGCGAAGCTTGAAGAT	R
**mmcrtYBF2-RT**	*crtYB* gene (*X. dendrorhous*)	TCGCATATTACCAGATCCATCTGA	F
**mmcrtYBR2-RT**		GGATATGTCCATGCGCCATT	R
**mmcrtIF-RT**	*crtI* gene (*X. dendrorhous*)	CATCGTGGGATGTGGTATCG	F
**mmcrtIR-RT**		GGCCCCTGATCGAATCGATAA	R
**mcrtSF-RT**	*crtS* gene (*X. dendrorhous*)	ATGGCTCTTGCAGGGTTTGA	F
**mcrtSR-RT**		TGCTCCATAAGCTCGATCCCAA	R
**mcrtRF-RT**	*crtR* gene (*X. dendrorhous*)	CTGGGAAACAAGACCTACGA	F
**mcrtRR-RT**		GGAACCTCGGTTACGACAAA	R
**HMGR_Real_2_F**	*HMGR* gene (*X. dendrorhous*)	GGCCGATCGCTATACATCCGTTT	F
**HMGR_Real_2_R**		ATCCAGTTGATGGCAGAAGGCT	R
**grg2real FW1**	*grg2* gene (*X. dendrorhous*)	CATCAAGACCTCTGTCACCAAC	F
**grg2real RV1**		TTGGCGTCAGACGAGGACT	R
**Inv real FW1**	*INV* gene (*X. dendrorhous*)	AGACCATCTGGCCTTCTCAAA	F
**Inv real RV1**		AGGACCGAAACTAGTGGATGTG	R
**pdcreal FW1**	*PDC* gene (*X. dendrorhous*)	TCAACACTGAGCTGCCCACT	F
**pdcreal RV1**		ATTCCGAATCGGGAAGCACA	R
**PDA1_F**	*PDA1* gene (*X. dendrorhous*)	GGATTCTGCCACTTGACGAT	F
**PDA1_R**		TCGGCAATAACTCCCTTCAC	R

EF-1α: 1α elongation factor; GPDH: glyceraldehyde-3-phosphate dehydrogenase. F: Forward; R: Reverse.
