# Supplementary material for: Regulation of carotenogenesis in the red yeast Xanthophyllomyces dendrorhous: the role of the transcriptional co-repressor complex Cyc8–Tup1 involved in catabolic repression
Source: Microb Cell Fact. 2016 Nov 14;15:193. doi: 10.1186/s12934-016-0597-1 (PMC5109733; doi:10.1186/s12934-016-0597-1)
Supplement: Supplementary file 2 — Additional file 2: Table S2. Carotenoid composition in X. dendrorhous wild-type and mutant strains cultured in MMv with 2% glucose. Analysis of carotenoid composition at different growth phases in X. dendrorhous wild-type, 385cyc8 − and 385tup1 − strains. [file 12934_2016_597_MOESM2_ESM.pdf]

Table S2. Carotenoid composition in *X. dendrorhous* wild-type and mutant strains cultured in MMv with 2% glucose.

Strain	Carotenoid	Growth curve phase				
		S1	S2	S3	S4	S5
**Wild-type**	Astaxanthin	79,2±9,4	76,1±7,6	67,9,2±3,6	69,7±8,0	71,0±2,1
	Beta-carotene	11,2±6,0	3,9±2,0	7,6±2,6	3,4±1,3	6,4±3,1
	Early intermediaries	ND	2,7±0,6	10,9±0,8	8,7±1,0	8,1±0,5
	Late intermediaries	10,4±0,7	16,1±2,7	16,7±2,8	15,4±1,4	16,1±1,3
**385*tup1*$^-$**	Astaxanthin	95,4±1,8 *	86,7±1,8 *	80,9±0,4 *	82,1±5,9 *	79,8±4,0 *
	Beta-carotene	ND	4,5±0,3	6,3±1,0	4,0±0,8	6,4±1,9
	Early intermediaries	4,6±1,8 **	ND *	5,7±1,2 **	6,9±1,2	6,6±0,6
	Late intermediaries	ND **	8,8±1,6 *	7,1±0,6 *	5,0±1,6 **	7,1±2,1 **
**385*cyc8*$^-$**	Astaxanthin	68±3,5	71,2±8	74,7±2,2	83,5±2,5*	86,5±3,2*
	Beta-carotene	11,4±1,7	8,27±1,2*	13±2,5*	6,6±0,6*	4,8±0,5
	Early intermediaries	14,8±4,1**	8,5±1**	9±0,2*	8,1±1,4	6,4±2,3
	Late intermediaries	4,4±1,2*	2,2±0,6**	3,5±1,5*	1,7±0,5**	2,3±0,4**

Early intermediaries: monocyclic carotenoids preceding beta-carotene in the synthesis pathway. Late intermediaries: carotenoids produced in the steps leading to astaxanthin from beta-carotene. Values are the percentage of each compound respect to the total carotenoid content and correspond to the average of three independent cultures and errors to the standard deviation. *: $p < 0,05$; **: $p < 0,01$, Student's t-test (Each mutant strain compared with respect to the wild-type strain). ND: not detected.
