# Supplementary figures and images for: Regulation of carotenogenesis in the red yeast Xanthophyllomyces dendrorhous: the role of the transcriptional co-repressor complex Cyc8–Tup1 involved in catabolic repression
Source: Microb Cell Fact. 2016 Nov 14;15:193. doi: 10.1186/s12934-016-0597-1 (PMC5109733; doi:10.1186/s12934-016-0597-1)

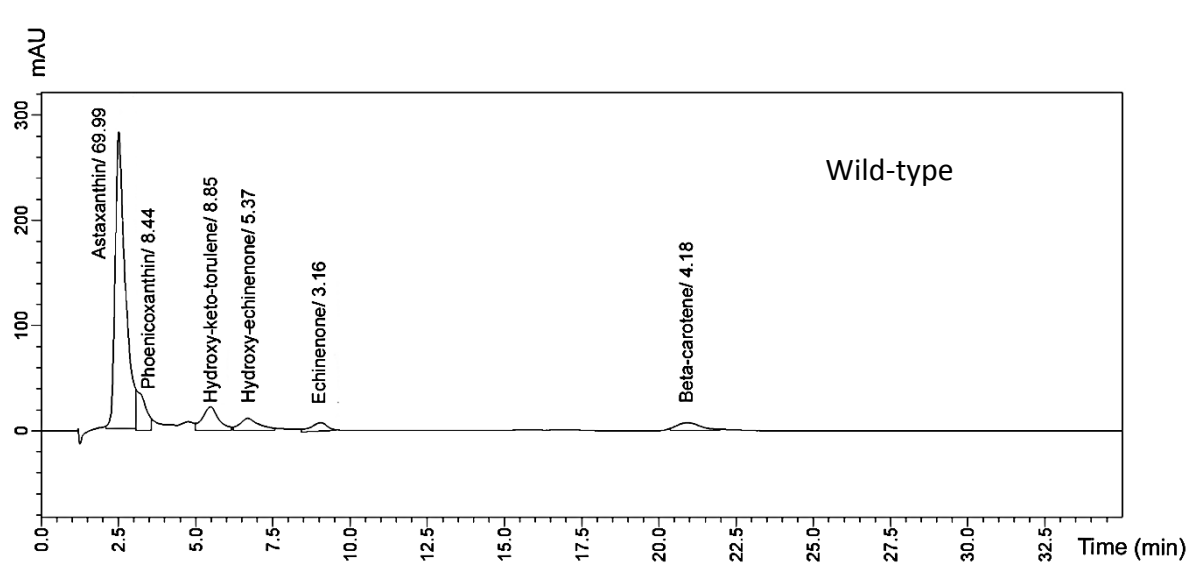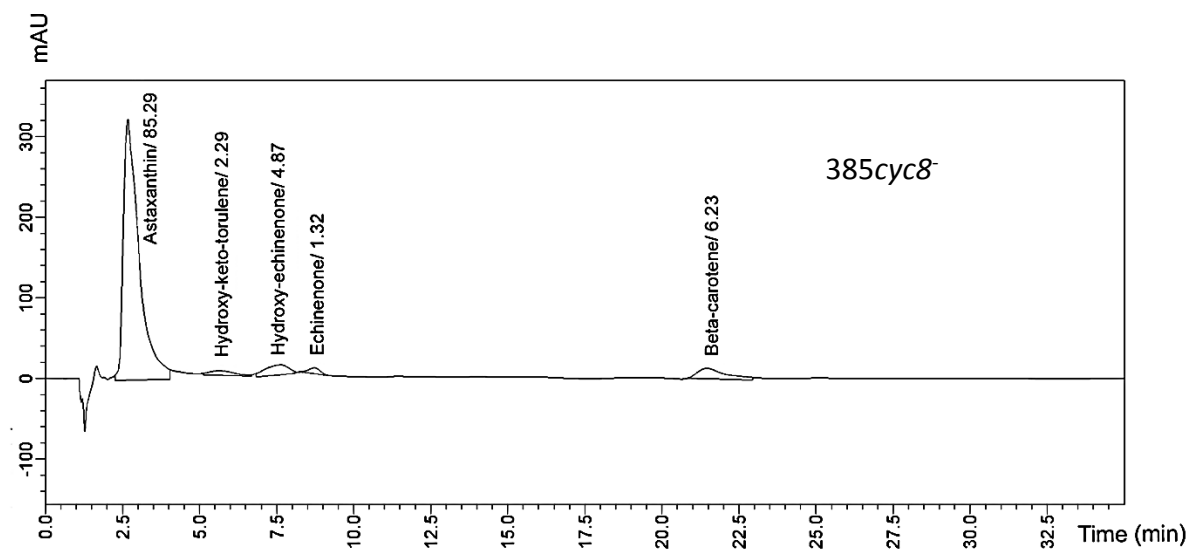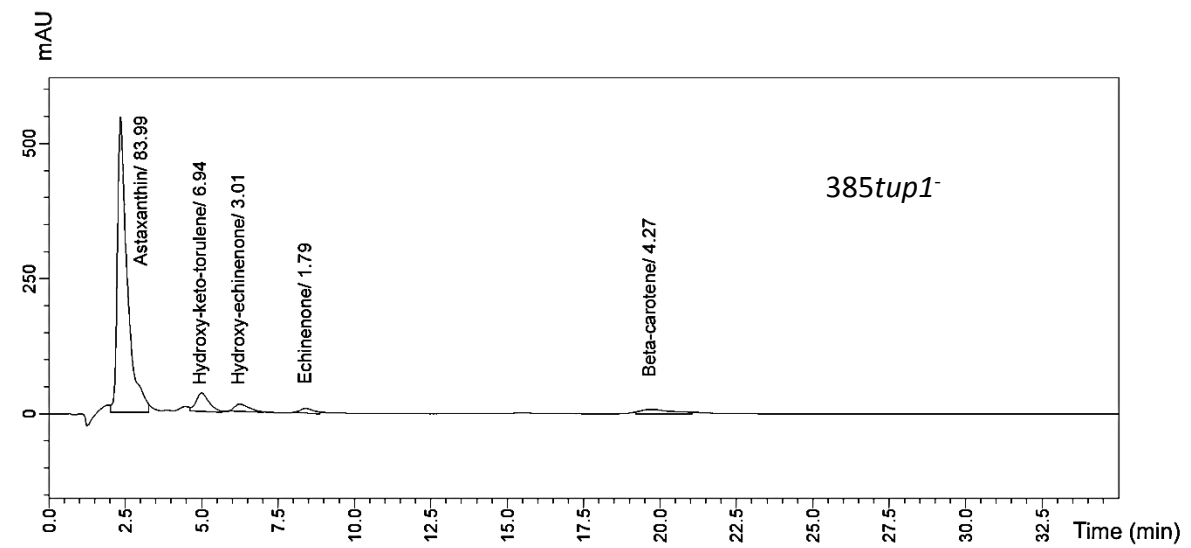

Supplement: Supplementary file 3 — Additional file 3: Figure S1. HPLC analysis of carotenoid composition of X. dendrorhous wild-type, 385cyc8 − and 385tup1 − strains. For each strain, a representative sample obtained at the late stationary phase of growth is shown. Values are the percentage representativeness of each compound with respect to the total carotenoid content. [file 12934_2016_597_MOESM3_ESM.pdf]

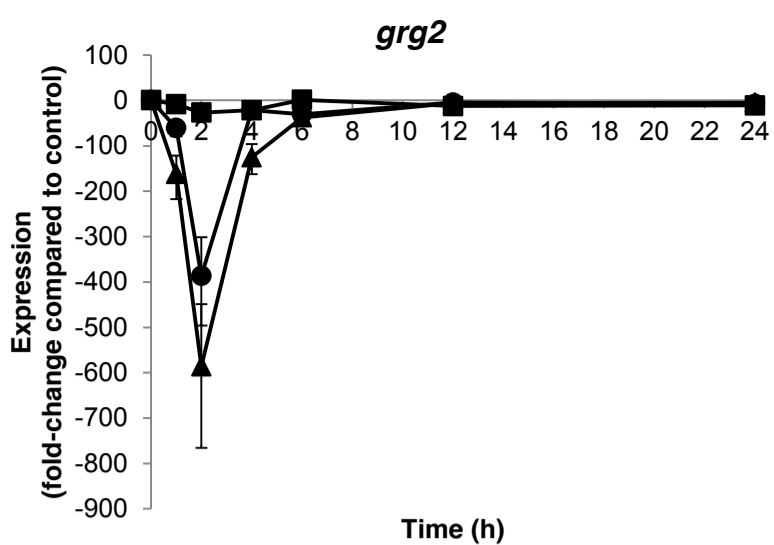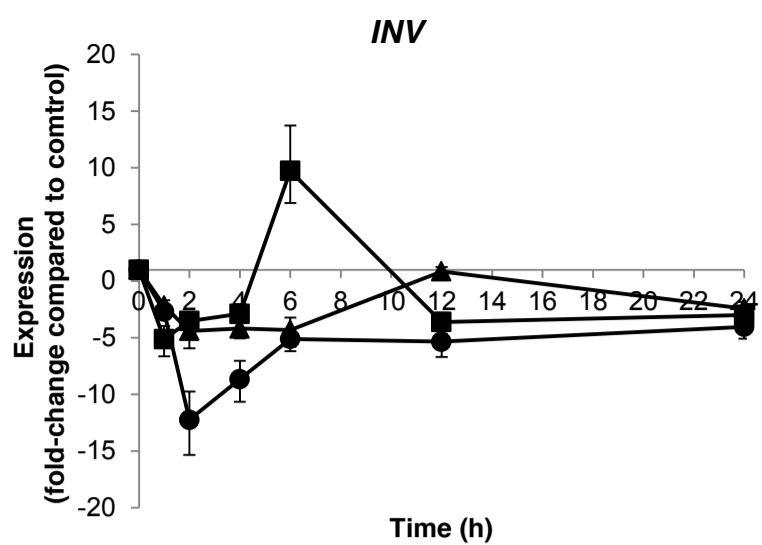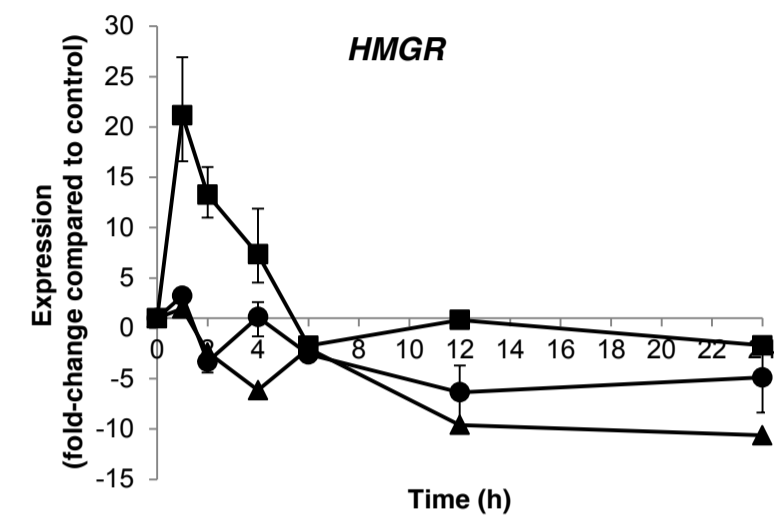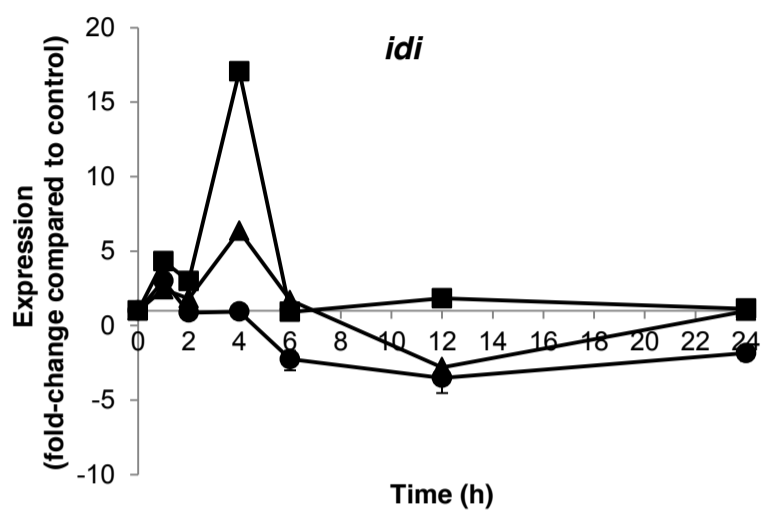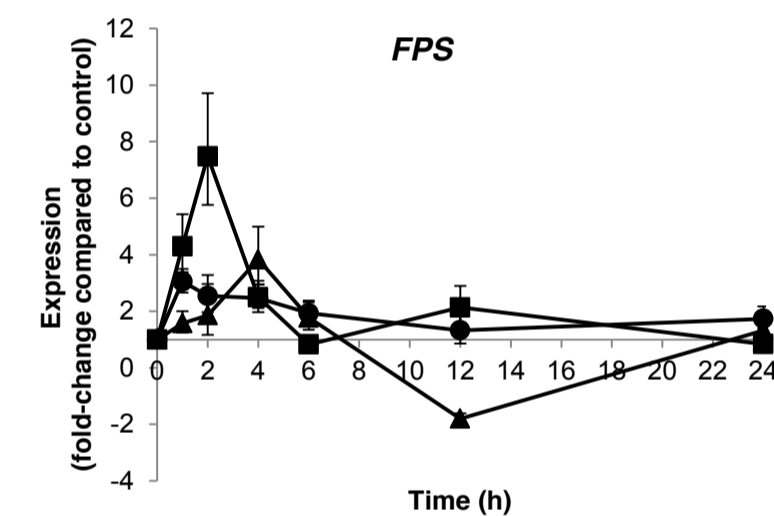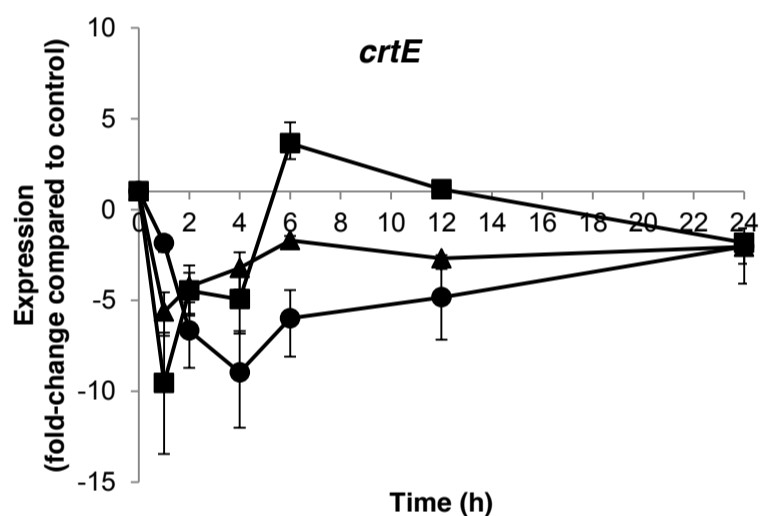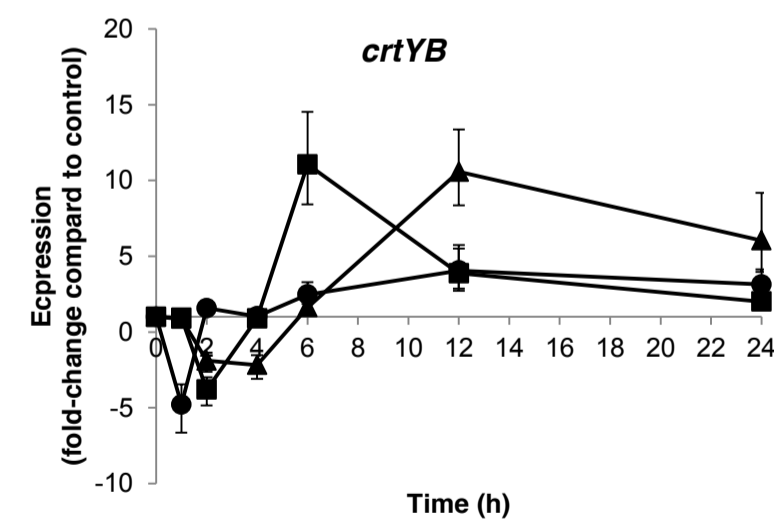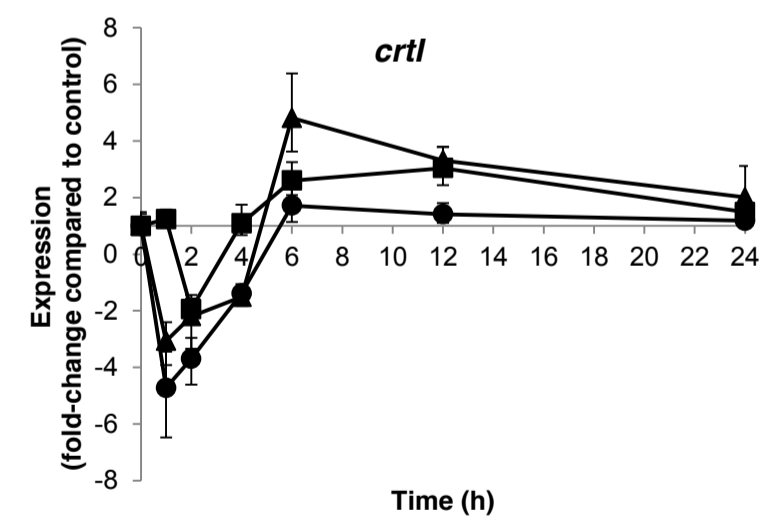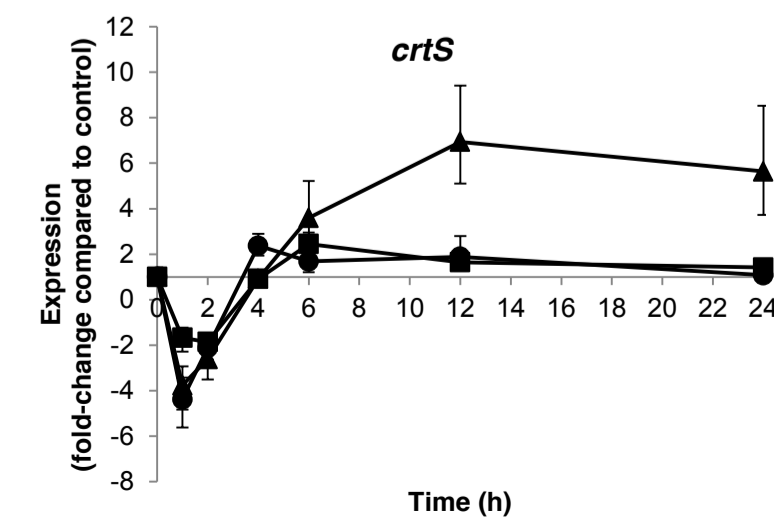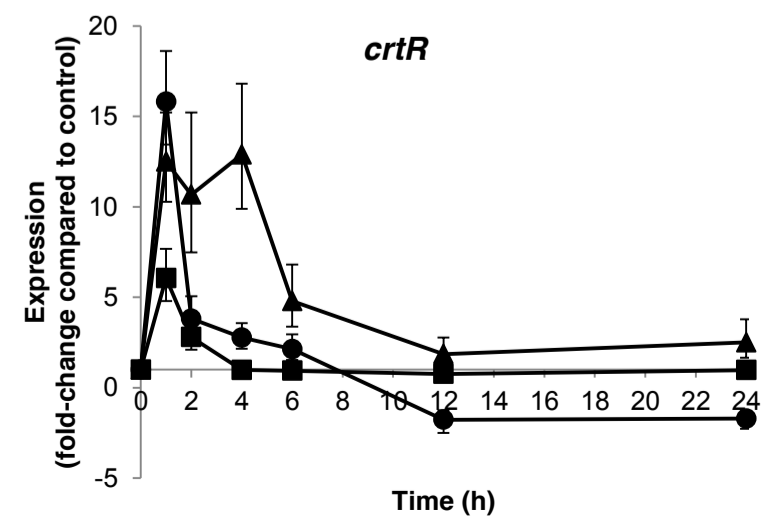

● Wild-type    ■ 385cyc8    ▲ 385tup1-

Supplement: Supplementary file 4 — Additional file 4: Figure S2. Glucose effect on transcript levels of genes involved in carotenogenesis. The expression kinetics at the mRNA level after the addition of glucose (final concentration of 20 g l−1) was determined relative to control (without glucose) for different genes involved in carotenogenesis (early stages: HMGR, idi, FPS and crtE; final stages: crtI, crtYB, crtS and crtR) and genes used as controls (INV and grg2) in the X. dendrorhous wild-type, 385cyc8 − and 385tup1 − strains. The horizontal axis of the graphs was set at 1. The data represent the average of three independent experiments, and the bars represent the standard error. [file 12934_2016_597_MOESM4_ESM.pdf]

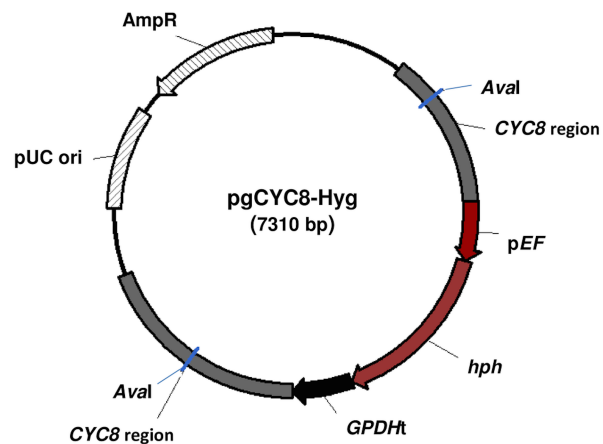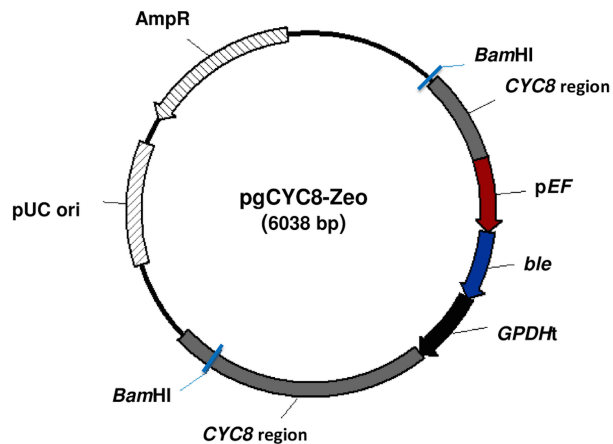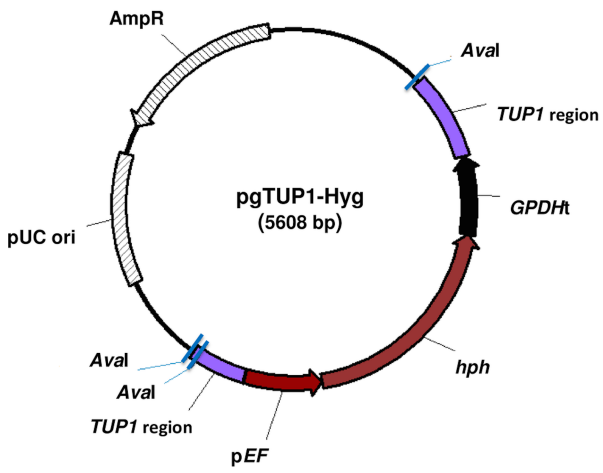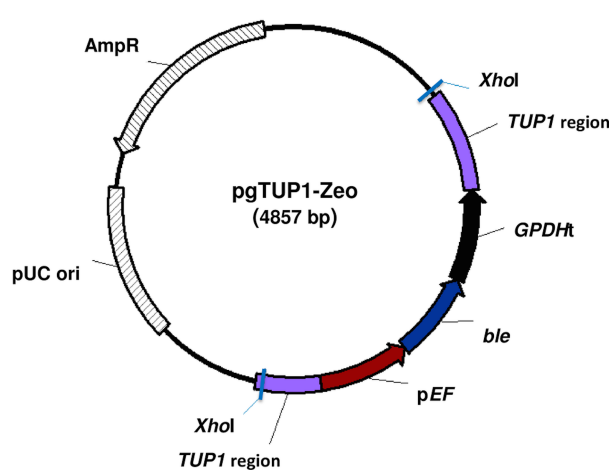

Supplement: Supplementary file 7 — Additional file 7: Figure S3. Plasmids used for X. dendrorhous transformation. Up: pgCYC8-Hyg and pgCYC8-Zeo plasmids used for 385cyc8 − mutant strain construction. Down: pgTUP1-Hyg and pgTUP1-Zeo plasmids used for 385tup1 − mutant strain construction. The restriction sites used to release the transformant DNA are shown. pEF: elongation factor 1α promoter (X. dendrorhous); GPDHt: glyceraldehyde 3 phosphate dehydrogenase terminator (X. dendrorhous). hph: hygromycin phosphotransferase gene (E. coli); ble: ble gene (Streptoalloteichus hindustanus); AmpR: ampicillin resistance; pUC ori: pUC replication origin. [file 12934_2016_597_MOESM7_ESM.pdf]
