# Supplementary material for: Regulation of carotenogenesis in the red yeast Xanthophyllomyces dendrorhous: the role of the transcriptional co-repressor complex Cyc8–Tup1 involved in catabolic repression
Source: Microb Cell Fact. 2016 Nov 14;15:193. doi: 10.1186/s12934-016-0597-1 (PMC5109733; doi:10.1186/s12934-016-0597-1)
Supplement: Supplementary file 5 — Additional file 5: Table S3. Identified overexpressed DEGs (according to the DESeq 2 results) in strain 385cyc8 −. Results of the BLAST analysis of the ORF sequences identified as overexpressed DEGs in strain 385cyc8 −. [file 12934_2016_597_MOESM5_ESM.pdf]

Table S3. Identified overexpressed DEGs (according to the DESeq2 results) in strain 385*cyc8*$^{-}$.

		BLAST results			
**ID**	**Potential gene product**	**Biological process**	**Organism**	**E-value**	**Identity (%)**
KX517841	MFS multidrug resistance transporter	Transmembrane transport	*Fusarium fujikuroi*	1.8 E-76	62
KX517842	Drug: H+ antiporter	Transmembrane transport	*Cryptococcus gattii*	2.0 E-122	44
KX517843	High affinity glucose transporter ght2	Transmembrane transport	*Rhizoctonia solani*	1.8 E-146	53
KX517844	Oligopepetide transporter OPT/MTD1	Transmembrane transport	*Rhodosporidium toruloides*	2.2 E-98	61
KX517845	OPT oligopeptide transporter	Transmembrane transport	*Rhizoctonia solani*	0.0	53
KX517846	AAT family aminoacid transporter	Transmembrane transport	*Laccaria bicolor*	3.0 E-83	54
KX517847	Neutral amino acid transporter	Transmembrane transport	*Aspergilluc flavus*	2.1 E-128	44
KX517848	Aminoacid permease	Transmembrane transport	*Punctularia strigosozonata*	9.3 E-28	38
KX517849	MFS transporter/Nicotinic acid transporter Tna1	Transmembrane transport	*Rhizoctonia solani*	5.3 E-122	44
KX517850	Major facilitator superfamily transporter/ Pantotenate transporter	Transmembrane transport	*Penicillium roqueforti*	0.0	69
KX517851	MFS general substrate transporter	Transmembrane transport	*Colletotrichum fioriniae*	5.8 E-19	48
KX517852	MFS general substrate transporter	Transmembrane transport	*Auricularia delicata*	0.0	57
KX517853	MFS general substrate transporter	Transmembrane transport	*Cryptococcus neoformans*	1.7 E-139	62
KX517854	MFS general substrate transporter	Transmembrane transport	*Melanopsichium pennsylvanicum*	6.3 E-139	47
KX517855	MFS protein/DUF895 domain membrane protein	Transmembrane transport	*Rhodosporidium toruloides*	7.6 E-15	41
KX517856	Mitochondrial carrier protein, tricarboxylate/iron carrier	Transmembrane transport	*Cryptococcus neoformans*	1.8 E-125	57
KX384930	Siderophore-iron transporter Str1	Transmembrane transport/ iron homeostasis	*Cryptococcus gattii*	4.0 E-95	70
KX517857	MOSC domain containing protein/ molybdenum cofactor sulfurase	Transmembrane transport	*Pseudozyma antarctica*	8.3 E-53	35
KX384901	PLP-dependent transferase/aminotransferase	Amino acid metabolism	*Fomitiporia mediterranea*	1.5 E-109	43
KX517858	GNAT family acetyltransferase/Acyl CoA acyltransferase	Protein/Histone modification	*Rhizoctonia solani*	3.0 E-50	40
KX517859	Acyl CoA N acyltransferase,GNAT family	Protein/Histone modification	*Glarea lozoyensis*	3.14 E-20	33
KX517860	Acyl CoA N acyltransferase	Protein modification	*Punctularia strigosozonata*	7.5 E-41	38
KX517861	Glycosyl transferase family 8 protein/ glycogenin	Protein modification	*Gloeophyllum trabeum*	4.9 E-98	50
KX517862	GNAT family acetyltransferase/Acyl CoA N-acyltransferase	Protein/Histone modification	*Rhizoctonia solani*	1.5 E-49	40
KX517863	Aspartic peptidase, endopeptidase	Proteolysis	*Cryptococcus neoformans*	1.6 E-60	42
KX517864	Imidazoleglycerol phosphate synthase/HisHF	Amino acid biosynthesis	*Cryptococcus gattii*	0.0	63

KX517865	Polisaccharide lyase family 1 protein/pectin lyase	Polysaccharide catabolic process	*Bipolaris zeicola*	1.8 E-22	69
KX517904	Six hairpin glycosidase/glycoside hydrolase family 125 protein	Carbohydrate metabolism	*Penicillium italicum*	8.9 E-115	44
KX517866	Glycoside hydrolase family 16 protein	Carbohydrate metabolism	*Rhizoctonia solani*	0.0	57
KX517867	Phosphoenolpyruvate carboxykinase	Gluconeogenesis	*Cryptococcus gattii*	0.0	83
KX517868	Fructose biphosphate aldolase	Glycolysis	*Rhizoctonia solani*	0.0	79
KX517869	2,4-dihydroxyhept-2-ene-1,7-dioic acid aldolase	phenylacetate catabolism	*Aspergillus kawachii*	5.4 E-144	75
KX384910	Alpha -beta hydrolase	Lipid metabolism	*Heterobasidion irregulare*	5.1 E-81	42
KX517870	SGNH hydrolase/ GDSL like lipase	Lipid metabolism	*Auricularia subglabra*	1.0 E-51	44
KX384927	Mevalonate kinase	Isoprenoid metabolism	*Cryptococcus gattii*	0.0	60
KX517871	Nitrilase	Nitrogen compound metabolism	*Paxillus involutus*	4.0 E-74	48
KX517872	NADP-dependent dehydrogenase acting on 3-hydroxy acids/malonic semialdehyde reductase	Nitrogen utilization/pyrimidine nucleobase catabolism	*Piriformospora indica*	7.4 E-110	59
KX517873	2-nitropropane dioxygenase	Pyrimidine nucleobase biosynthesis	*Cryptococcus gattii*	1.0 E-64	72
KX517874	2-Oxoglutarate-Fe (II) dependent oxygenase	DNA repair	*Trametes versicolor*	2.8 E-31	48
KX517875	DNA repair protein rev1	DNA repair	*Moniliophtora roreri*	9.0 E-5	40
KX517876	Transcriptional regulator	Transcription regulation	*Cryptococcus gattii*	1.4 E-97	59
KX517877	PacC, Ph response transcription factor	Transcription regulation	*Rhizoctonia solani*	0.0	43
KX517878	HMG-box transcription factor/mating type protein MAT1-2	Transcription regulation	*Melanopsichium pennsylvanicum*	8.1 E-10	35
KX517879	Nitrogen assimilation transcription factor/ nit-4	Transcription regulation	*Rhodosporidium toruloides*	1.4 E-9	32
KX517880	Stress response protein rds1p	Stress response	*Piriformospora indica*	1.1 E-84	54
KX517881	Sporulation specific protein spo7	Sporulation/stress response	*Postia placenta*	2.7 E-27	49
KX517882	Meiotic recombination protein SPO11	Sporulation/ stress response	*Cryptococcus gattii*	1.3 E-4	50
KX517883	RIO/RIO1 protein kinase	Cell cycle and Ribosome biogenesis	*Rhizoctonia solani*	5.64 E-9	64
KX384909	G1/S specific cyclin Pcl1	Cell cycle	*Cryptococcus neoformans*	4.0 E-61	49

ID: accession number of the identified DEG
