# Supplementary material for: Regulation of carotenogenesis in the red yeast Xanthophyllomyces dendrorhous: the role of the transcriptional co-repressor complex Cyc8–Tup1 involved in catabolic repression
Source: Microb Cell Fact. 2016 Nov 14;15:193. doi: 10.1186/s12934-016-0597-1 (PMC5109733; doi:10.1186/s12934-016-0597-1)
Supplement: Supplementary file 6 — Additional file 6: Table S4. Identified overexpressed DEGs (according to the DESeq 2 results) in strain 385tup1 −. Results of the BLAST analysis of the ORF sequences identified as overexpressed DEGs in strain 385tup1 −. [file 12934_2016_597_MOESM6_ESM.pdf]

Table S4. Identified overexpressed DEGs (according to the DESeq2 results) in strain 385*tup1*[-].

		BLAST results			
**ID**	**Potential gene product**	**Biological process**	***Organism***	**E-value**	**Identity (%)**
KX517884	MFS general substrate transporter/drug resistance	Transmembrane transport	*Metarhizium guizhouense*	2.6 E-127	41
KX517885	Auxin efflux carrier	Transmembrane transport	*Cryptococcus gattii*	2.0 E-80	44
KX517886	Vacuolar protein sorting 55	Intracellular protein transport	*Pseudozyma hubeiensis*	3.0 E-49	65
KX517887	Peroxisomal membrane protein 4	Intracellular protein transport/ protein import into peroxisome	*Rhizoctonia solani*	4.7 E-90	59
KX517888	Peroxin 14	Intracellular protein transport/ protein import into peroxisome	*Cryptococcus gattii*	9.0 E-31	38
KX517889	Translocator protein/TspO/MBR like protein	Mitochondrial transmembrane transport	*Rhizoctonia solani*	2.6 E-31	56
KX517890	Alpha,alpha trehalase/glycoside hydrolase family 37 protein	Carbohydrate metabolic process/cellular response to desiccation	*Rhizoctonia solani*	0.0	51
KX517891	Phosphoketolase	Carbohydrate metabolic process	*Cryptococcus gattii*	0.0	63
KX517892	Trehalose synthase	Carbohydrate metabolic process/stress response	*Cryptococcus neoformans*	0.0	56
KX517905	Glycoside hydrolase family 29 protein/alpha L fucosidase	Carbohydrate metabolic process	*Auricularia delicata*	6.6 E-28	40
KX517893	NADP-dependent alcohol dehydrogenase	Alcohol metabolism	*Gloeophyllum trabeum*	5.2 E-141	63
KX517894	Zinc type-alcohol dehydrogenase/butanediol dehydrogenase	Alcohol metabolism	*Punctularia strigosozonata*	0.0	70
KX517895	Coproporphyrinogen III oxidase	Heme biosynthetic process	*Gloeophyllum trabeum*	7.2 E-128	54
KX517896	Isochorismatase hydrolase	Siderophore group biosynthetic process	*Trametes versicolor*	5.1 E-56	50
KX517897	C-4 methyl sterol monooxygenase	Ergosterol biosynthetic process	*Rhizoctonia solani*	1.4 E-126	58
KX517898	G/T mismatch specific DNA glycosylase	DNA repair	*Gloeophyllum trabeum*	8.9 E-45	41
KX517899	Universal stress protein/ A family	Stress response	*Pseudozyma brasiliensis*	7.7 E-26	33
KX517900	S-adenosyl-L-methionine-dependent methyltransferase	Protein/Lipid/nucleic acid modification	*Punctularia strigosozonata*	2.0 E-40	39
KX517901	Metallo-hydrolase oxidoreductase	tRNA processing	*Fomitiporia mediterranea*	4.7 E-106	42

ID: accession number of the identified DEG
